# Effects of tail nerve electrical stimulation on the activation and plasticity of the lumbar locomotor circuits and the prevention of skeletal muscle atrophy after spinal cord transection in rats

**DOI:** 10.1111/cns.14445

**Published:** 2023-09-26

**Authors:** Jia‐Lin Liu, Zheng‐Hong Chen, Rong‐Jie Wu, Hai‐Yang Yu, Shang‐Bin Yang, Jing Xu, Chuang‐Ran Wu, Yi‐Nan Guo, Nan Hua, Xiang Zeng, Yuan‐Huan Ma, Ge Li, Ling Zhang, Yuan‐Feng Chen, Yuan‐Shan Zeng, Ying Ding, Bi‐Qin Lai

**Affiliations:** ^1^ Key Laboratory for Stem Cells and Tissue Engineering (Sun Yat‐sen University), Ministry of Education Guangzhou Guangdong China; ^2^ Rehabilitation Medicine Department The First Affiliated Hospital of Sun Yat‐sen University Guangzhou Guangdong China; ^3^ Shantou University Medical College Shantou Guangdong China; ^4^ Department of Orthopedics Guangdong Provincial People's Hospital, Guangdong Academy of Medical Sciences Guangzhou Guangdong China; ^5^ Department of Histology and Embryology Zhongshan School of Medicine, Sun Yat‐sen University Guangzhou Guangdong China; ^6^ Guangdong Provincial Key Laboratory of Brain Function and Disease Zhongshan School of Medicine, Sun Yat‐sen University Guangzhou Guangdong China; ^7^ Guangzhou First People's Hospital, Guangzhou Institute of Clinical Medicine, South China University of Technology Guangzhou Guangdong China; ^8^ Guangdong Provincial Key Laboratory of Pathogenesis, Targeted Prevention and Treatment of Heart Disease Guangdong Provincial People's Hospital(Guangdong Academy of Medical Sciences), Southern Medical University Guangzhou Guangdong China; ^9^ Co‐innovation Center of Neuroregeneration Nantong University Nantong Jiangsu China

**Keywords:** motor neural circuit activation, muscle atrophy, regeneration, spinal cord transection, tail nerve electrical stimulation

## Abstract

**Introduction:**

Severe spinal cord injury results in the loss of neurons in the relatively intact spinal cord below the injury area and skeletal muscle atrophy in the paralyzed limbs. These pathological processes are significant obstacles for motor function reconstruction.

**Objective:**

We performed tail nerve electrical stimulation (TNES) to activate the motor neural circuits below the injury site of the spinal cord to elucidate the regulatory mechanisms of the excitatory afferent neurons in promoting the reconstruction of locomotor function.

**Methods:**

Eight days after T10 spinal cord transection in rats, TNES was performed for 7 weeks. Behavioral scores were assessed weekly. Electrophysiological tests and double retrograde tracings were performed at week 8.

**Results:**

After 7 weeks of TNES treatment, there was restoration in innervation, the number of stem cells, and mitochondrial metabolism in the rats' hindlimb muscles. Double retrograde tracings of the tail nerve and sciatic nerve further confirmed the presence of synaptic connections between the tail nerve and central pattern generator (CPG) neurons in the lumbar spinal cord, as well as motor neurons innervating the hindlimb muscles.

**Conclusion:**

The mechanisms of TNES induced by the stimulation of primary afferent nerve fibers involves efficient activation of the motor neural circuits in the lumbosacral segment, alterations of synaptic plasticity, and the improvement of muscle and nerve regeneration, which provides the structural and functional foundation for the future use of cutting‐edge biological treatment strategies to restore voluntary movement of paralyzed hindlimbs.

## Graphical abstract

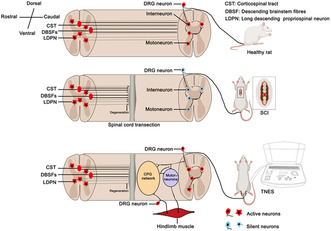

## 1 INTRODUCTION

Transected spinal cord injury (SCI) may induce the loss of innervation in the spinal cord below the injury area, thus resulting in the loss of locomotor function.^1^ Although spinal cord tissue below the level of the injured segments may be relatively intact, the long‐term loss of excitatory input from the brain can lead to functional silencing of neurons, cell body atrophy, and apoptosis.^2^, ^3^, ^4^ These processes can subsequently develop into irreversible atrophy of muscles in the paralyzed limbs.^5^, ^6^ Hence, these pathological processes are considered crucial obstacles in the reconstruction of locomotor function.

At present, the treatment for SCI focuses on cell transplantation and tissue engineering, both of which aim to promote regeneration of the corticospinal tract (CST) and other brain‐derived nerve axons or facilitate the formation of new neural circuits at the injury site to relay neural information and eventually restore innervation to the paralyzed limbs.^7^, ^8^, ^9^ However, even after decades of research, there has been no breakthrough in locomotor function recovery. An exciting development was the recent research from Courtine et al, who reported that electrical stimulation of the spinal cord below the level of the injured segments could induce limb movement.^10^ Subsequently, the researchers conducted a series of studies on the brain–computer interface and spinal cord epidural electrical stimulation (EES) and demonstrated that electrical stimulation of the spinal cord at a certain frequency could re‐initiate limb movement in the absence of inputs from the brain.^11^, ^12^, ^13^ However, the implementation of these programs requires the prevention of neuronal apoptosis and alleviation of atrophy in the muscles of the paralyzed limbs.

Both EES and direct intra‐spinal micro‐stimulation (ISMS) at the lumbosacral spinal cord have been proven to induce walking and produce electromyograms (EMGs) after high‐level SCI.^12^, ^14^, ^15^ These treatments act via the central pattern generator (CPG).^4^, ^6^, ^16^ However, the application of EES and ISMS requires electrode implantation; these techniques produce certain side effects and can only be applied in certain circumstances, thus limiting their clinical application.^17^, ^18^ Therefore, it is of great significance to identify non‐invasive, safe and effective stimulation protocols that can activate the CPG and motor neural circuits in the spinal cord.

In a previous study, we found that medium‐frequency electrical stimulation of the tail root of rats with transection SCI could effectively protect neurons in the L1–L5 segment of the spinal cord and prevent muscle atrophy in the hindlimbs.^6^ Other studies have shown that the application of tail nerve electrical stimulation (TNES) in rats with spinal cord contusion could promote the recovery of spontaneous locomotor function.^19^ However, the specific nerve conduction pathway involved in this process was unclear; thus, there is an urgent need for detailed investigations relating to the mechanisms underlying TNES. Accordingly, in the present study, we focused on the mechanisms of TNES in regulating the plasticity of the motor neural circuits to improve skeletal muscle atrophy and promote locomotor function reconstruction in a rat model of SCI. The effects of TNES in rats are likely to represent neuroregulatory and biological responses that also exist in humans.^17^, ^20^ Therefore, further studies on the mechanisms by which motor neural circuits can be activated and the improvement of skeletal muscle atrophy by TNES can provide significant theoretical support for the development of new clinical treatments for SCI.

## 2 METHODS

### 2.1 Surgery

All the experimental protocols and animal handling procedures were approved by the Animal Care and Use Committee of Sun Yat‐sen University and were consistent with the National Institutes of Health Guide for the Care and Use of Laboratory Animals (approval reference number: SYSU‐IACUC‐2019‐B1101). An intraperitoneal injection of 1% pentobarbital sodium (25–35 mg/kg, China Pharmaceutical Shanghai Chemical Reagent Company) was used to induce anesthesia during total transection surgery. First, the skin was disinfected and the body was fixed. Then, the skin and superficial fascia were cut under aseptic conditions. The surgical area was fixed with self‐made hooks and the muscles and ligaments were bluntly separated to clearly expose the T9 spinous process and vertebral arch. Next, we used a bone‐ribbing rongeur to gently clamp out the vertebral arch root along the intervertebral space between T9 and T10 and gradually clamp off the T9 vertebral arch to expose the T10 spinal cord. Then, we cut the spinal dura mater, which was completely transected and rapidly transected 2 mm from the incision. The spinal cord in the middle of the two cuts was extracted using pointed forceps. Finally, we sequentially sutured the muscle layer, subcutaneous tissue, and skin. After surgery, each rat was injected subcutaneously with 0.05 mg/kg of buprenorphine (Tianjin Pharmaceutical Research Institute Pharmaceutical Co., Ltd.) every 12 h for 3 consecutive days to relieve pain; intramuscularly with penicillin G (50,000 U/kg/d, Jiangxi Keda Animal Pharmaceutical Co., Ltd.) for 5 days; and manual emiction twice a day after surgery.

### 2.2 TNES treatment

Rats were divided into four groups after surgery (*n* = 10 rats/group), and each group received treatment starting 1 week after surgery. Rats in the TNES group were placed in a square box (side length: 1 m) and two electrodes coated with conductive adhesive were placed 1 cm apart on the root of the tail (Video S1). We selected prescription 8 in an intermediate frequency instrument (Type J18A1, Quan‐Ri‐Kang Company); electrical stimulation was given while the rats were awake and freely moving, and there was no struggle or screaming during stimulation. The output frequency was 4 kHz, and the stimulation intensity was adjusted from 10 to 30 mA to observe slight skeletal muscle contraction and slight movement in one joint or two joints in the paralyzed hindlimbs (Video S2). The treatment lasted 20 min on each occasion and was repeated five times a week for 7 weeks. Rats in the sham TNES group were treated in the same manner, but with the electrical stimulator turned off. In addition, electrodes were placed on the muscle belly of the tibialis anterior and gastrocnemius muscles of the hindlimbs in the SkMES group; the treatment parameters were the same as those used for the TNES group (prescription 8, 4 kHz). The stimulation intensity was also adjusted from 10 to 30 mA to observe slight skeletal muscle contraction and slight movement in one joint or two joints in the paralyzed hindlimbs. Rats in the SCI group received no treatment, and the feeding conditions and detection indices were the same as those of the other group.

### 2.3 Assessment of behavioral recovery

Rats in each group were subjected to tests for hindlimb function every week after surgery. For the Basso, Beattie, and Bresnahan (BBB) open‐field locomotor test,^21^ rats were allowed to crawl freely in a square box (width: 1 m) for 5 min. We mainly observed the frequency and range of movement of the three joints (hip, knee, and ankle) in the hindlimbs and whether the hindlimbs could support the body and coordinate with the forelimbs (Video [Link], [Link], [Link], [Link], and S11). During the first week after surgery, we observed the behavior of the animals. Rats with BBB scores >3, with hindlimb spasticity, or with sensory abnormalities such as leg biting were excluded from the experiment.

In the grid climbing test, rats were placed on a grid with a 45° slope and their hindlimbs were observed and videotaped for 3 min^22^ (Video ) We mainly observed the overall movement ability of rats within a unit of time to climb over obstacles and whether there was coordinated movement between the forelimbs and hindlimbs.

### 2.4 Nervous pathways tracing

Eight weeks after SCI, the rats were subjected to abdominal anesthesia with 1% pentobarbital sodium (35 mg/kg). Next, we cut the skin along the femur, bluntly separated the muscle with scissors and hooked the general branch of the sciatic nerve. Then, a microneedle containing 1 μL of 3% FG (Fluorochrome) was inserted into the sciatic nerve under a microscope and left for 4 min after injection. Finally, we sutured the tissues layer by layer. For tail nerve tracing, 2 μL of 2% cholera toxin B (CTB) conjugated to Alexa Fluor 555 (Molecular Probes) was injected into multiple injection sites in the tail nerve (0.1 μL per each injection site). The rats were fed conventionally for 1 week after injection.

### 2.5 Electrophysiology

Electrophysiological examination was performed on each experimental rat prior to perfusion. The rats were anesthetized by an injection of 1% pentobarbital sodium (30 mg/kg) and fixed with stereotaxic apparatus. The sensorimotor cortex and sciatic nerve were then exposed. Then, a silver ball‐guided reference electrode was placed on one side of the exposed sensorimotor cortex, a hooked recording electrode was placed on the contralateral sciatic nerve and a reference electrode was placed on the skin incision. Next, we used the NeuroExam M‐800 Data Acquisition Analysis System (MEDCOM) and selected “Experimental project—Central nervous experiment—cerebral cortex‐evoked potential” with a gain of 2000, a time constant of 0.01 s, a filter of 300 Hz, a delay of 50 ms, a voltage of 3 mV and a wave width of 0.1 ms. Cortical motor‐evoked potentials (CMEPs) were stimulated and recorded and 10 stable wave patterns were collected for each animal per test; mean values of their amplitude and latency were calculated.

### 2.6 Perfusion, frozen sections and wet weight of muscles

Rats in each group were perfused 8 weeks after SCI while rats injected with FG/CTB were perfused after 9 weeks. First, we injected 1% of a lethal dose of sodium pentobarbital (50 mg/kg) and cut open the chest wall at the xiphoid process to fully expose the heart. Next, a perfusion tube was inserted through the left ventricle into the ascending aorta and secured with hemostatic forceps. Then, we cut open the right atrial appendage at the beginning of the infusion and infused 200 mL of normal saline to flush away blood and added 300 mL of 4% paraformaldehyde to fix the tissue. After perfusion, the spinal cord tissues were placed in 4% paraformaldehyde at 4°C for 24 h followed by dehydration in 30% sucrose. Then, we cut 25 μm longitudinal sections and 30 μm transverse sections with a freezing microtome. All sections were stored in a refrigerator at −30°C for subsequent analysis. In addition, to determine wet weights, the gastrocnemius and tibialis anterior muscles of the hindlimbs were removed under an overdose of anesthesia and the wet muscle mass was determined with an electronic analytical balance with an accuracy of 0.1 g.

### 2.7 Protein extraction and western blot

The L1–S2 segments of the spinal cord and gastrocnemius muscle were weighed and a corresponding volume of cell lysis buffer (containing 4% protease inhibitor) was added at a ratio of 1 mg/10 μL. Next, the tissues were crushed by ultrasound and centrifuged at 12,000 rpm/min for 20 min at 4°C. Finally, the transparent protein layer in the middle was extracted and stored at 80°C for subsequent analysis.

Specific proteins were prepared with 5 × SDS–PAGE loading buffer and ddH_2_O. Electrophoresis was then performed at 80 V for 30 min and then the voltage was increased to 100 V for 70 min. Then, electrotransfer was performed at a low temperature with a constant current of 250 mA for 240 min. Membranes were then incubated overnight with specific primary antibodies at 4°C (Indicators relate to antibodies in the Table in Data S1). The next morning, the membranes were incubated with horseradish peroxidase (HRP)‐conjugated secondary antibodies for 2 h. Finally, membranes were placed into a ChemiDoc Touch (Bio‐RAD) imaging system for enhanced chemiluminescence (ECL) imaging. (Original blot images in Data S2).

### 2.8 Neutral red staining

Frozen sections from the L1 and L4 spinal cord were stained with neutral red. First, the sections were soaked in 1% neutral red dye for 25 min and dehydrated with 70%, 80%, 95%, and 100% alcohol for 5 s each. Then, the slides were cleared in van‐clear reagents I and II for 4 min. Finally, the slides were sealed with neutral gum, dried, and observed under a light microscope. To analyze the survival of neurons in the L1 dorsal nucleus and motoneurons in the anterolateral horn of the L4 segment, every 5th section of the spinal cord segment was collected. A total of 5 sections from each segment per rat were used for neuronal counts after neutral red staining (*n* = 5). The number of dorsal nucleus and motoneurons with intensely stained neutral red in the cytoplasm and well‐delineated nucleus of the right and left dorsal nucleus and ventral horns for each section was counted under a light microscope at the magnification of 200×. The number of neurons in two sides of each section was pooled to yield a total number of cells per section. Finally, the total number of neurons of 5 sections derived from each rat was presented for statistical analysis (*n* = 5).

### 2.9 Hematoxylin and eosin (HE) staining

Frozen sections of muscle were dehydrated with 95% alcohol for 5 min. Then, the sections were stained with hematoxylin for 10 min and decolorized with 1% hydrochloric acid alcohol for 20 s. Then, the sections were washed with tap water for 30 min and stained with eosin for 1 min. Next, the slides were sequentially dehydrated with 80%, 90%, 95%, and 100% alcohol and cleared twice in xylene for 30 min. Finally, the slides were sealed with neutral gum in a fume cupboard and observed under a light microscope. For quantification of the cross‐sectional area of myofiber, one section of every 100 μm of the selected muscle segment and a total 5 sections of each rat (*n* = 5 in each group) were collected for HE staining. Images of random fields of each muscle section were captured under a bright field microscope at a typical magnification of 200×. Myofiber area in the tibialis anterior muscles was determined using the ImageJ software (NIH).

### 2.10 Morphological analysis

For the quantification of c‐Fos^+^ and Pax7^+^ (paired box gene 7 protein, a marker of skeletal muscle stem cell) cells, one in every five sections from each animal was processed; a total of three sections per rat were analyzed (*n* = 5 in each group). The immunopositive cells was calculated by counting the total number of immunopositive cells in the selected fields. For quantification of activity‐regulated cytoskeleton associated protein (Arc), one in every five sections from each animal was processed, and a total of three sections per rat were analyzed (*n* = 5 in each group). The fluorescence density of the Arc^+^ area was calculated. For quantification of innervated motor endplates, synaptophysin positive (SYP^+^) motor endplates were divided by the total number of motor endplates in the selected fields. For quantification of the vesicular glutamate transporters 1 positive (VGluT1^+^) and glutamic acid decarboxylase 1 positive (GAD67^+^) presynaptic terminals on the surface of FG^+^ motoneurons, the gray value changes of VGluT1^+^ and GAD67^+^ presynaptic terminals on the surface of FG^+^ motoneurons were detected, and the number of curve peaks was included in the statistics; a total of three sections per rat were analyzed (*n* = 5 in each group). The steps of fluorescence curve analysis are as follows: the image was selected in ImageJ software. Next, the option “Segmented line” in the “Straight” tool was selected. Then, the specific fluorescence channel to be analyzed in the image (such as the fluorescence channel represented by VGluT1^+^ or GAD67^+^) was selected, and “Segmented line” was used to outline the part with fluorescence expression (VGluT1^+^ or GAD67^+^) on the surface of selected FG^+^ neurons. Finally, the “Plot profile” tool was selected in the “Analyze” option to generate a corresponding curve graph based on the change in fluorescence intensity of the part outlined.^23^ The ordinate indicates the fluorescence intensity value, and the abscissa indicates the distance from the beginning to the end of the line. The number of curve peaks represents the number of presynaptic terminals with VGluT1^+^ or GAD67^+^ on the surface of FG^+^ neurons.

To identify the afferent pathway that receives electrical stimulation from the tail nerve, CTB^+^ nerve fibers that formed contacts with the FG^+^ motor neurons or with the ephrin A4 receptor (EphA4) and VGluT2 double‐positive neurons (EphA4^+^/VGluT2^+^ CPG neurons) were observed in the L2 and L5 segments.^16^ For in vivo quantification of neurofilament (NF)^+^ axons, areas that were within 1 mm rostral or caudal of the SCI site or in the 1 mm injury site of each of the horizontal sections were chosen. One in every five sections from each animal was processed; a total of three sections per rat were analyzed (*n* = 5 in each group). NF^+^ nerve fibers, with a length greater than 20 μm in the selected fields, were counted. (Indicators relate to antibodies in the Table in Data S1).

### 2.11 Statistical analysis

All the statistical analyses were performed using the statistical software GraphPad Prism (v 8.0; GraphPad Software). Data are expressed as the mean ± standard error (SEM). When *n* < 50, the Shapiro–Wilk test was used to evaluate the data distribution. Otherwise, the Kolmogorov–Smirnov test was used. For normal distributions of variable values, two group comparisons were analyzed using unpaired *t* test and multiple group comparisons were analyzed using one‐way analysis of variance (anova) with Bonferroni post‐hoc correction. For non‐normally distributed variable values, Kruskal–Wallis test was used to analyze the differences between groups. The BBB score was analyzed with repeated measures and two‐way anova, followed by Tukey's post‐hoc test to compare the differences between groups on the same day. A statistically significant difference was accepted at *p* < 0.05.

## 3 RESULTS

### 3.1 Identification of lumbosacral spinal cord conduction pathway by TNES

**FIGURE 1:**
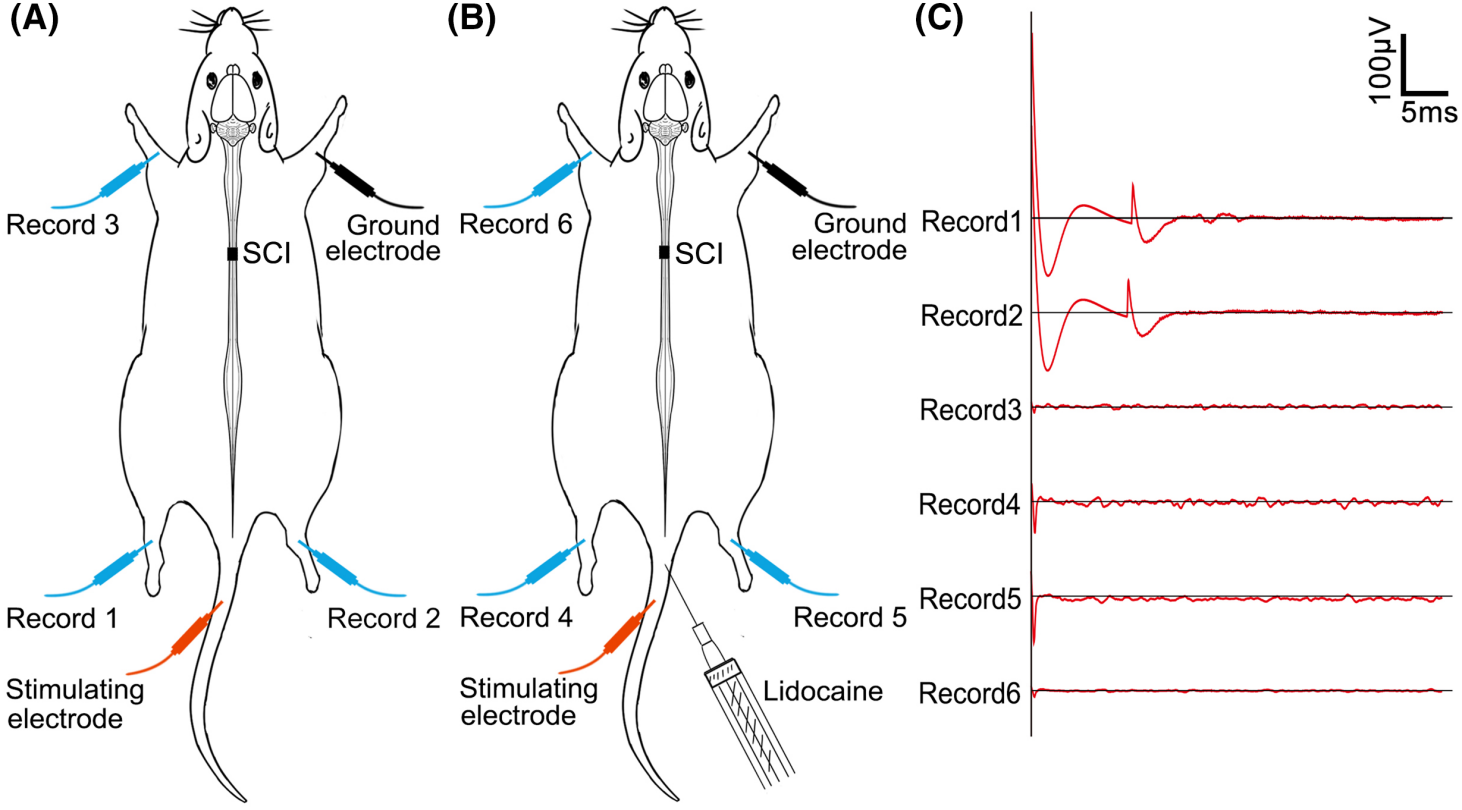
The neural pathway associated with tail nerve electrical stimulation (TNES). (A) EMGs could be recorded in the tibialis anterior muscles of both hindlimbs (record 1 and record 2) but no signals were recorded in the forelimbs (record 3) during TNES treatment. (B) When lidocaine was injected at multiple points around the tail root, EMGs could not be recorded in the tibialis anterior muscles of both hindlimbs (record 4 and record 5) or in the forelimbs (record 6) during TNES treatment.

To explore whether there was a spinal cord conduction pathway between the tail nerve and the skeletal muscles of the hindlimbs, we performed TNES under anesthesia in T10 transected SCI rats and recorded the EMGs of the tibialis anterior muscles in the hindlimbs (Figure 1A–C). When the tail nerve was stimulated (Figure 1A), action potentials were recorded in the tibialis anterior muscles of both hindlimbs; however, no EMG signal was recorded in the forelimbs (Figure 1C). Then, lidocaine was injected at multiple points around the tail root to block electrical signal transmission in the tail nerve (Figure 1B). We were unable to record the EMGs in the hindlimbs during the TNES treatment (Figure 1C). These results showed that electrical signals were transmitted in an afferent nerve manner through the tail nerve to the motor neural circuits of the lumbosacral spinal cord to affect muscular excitability in the hindlimbs.

### 3.2 The effect of TNES on hindlimb muscle atrophy

**FIGURE 2:**
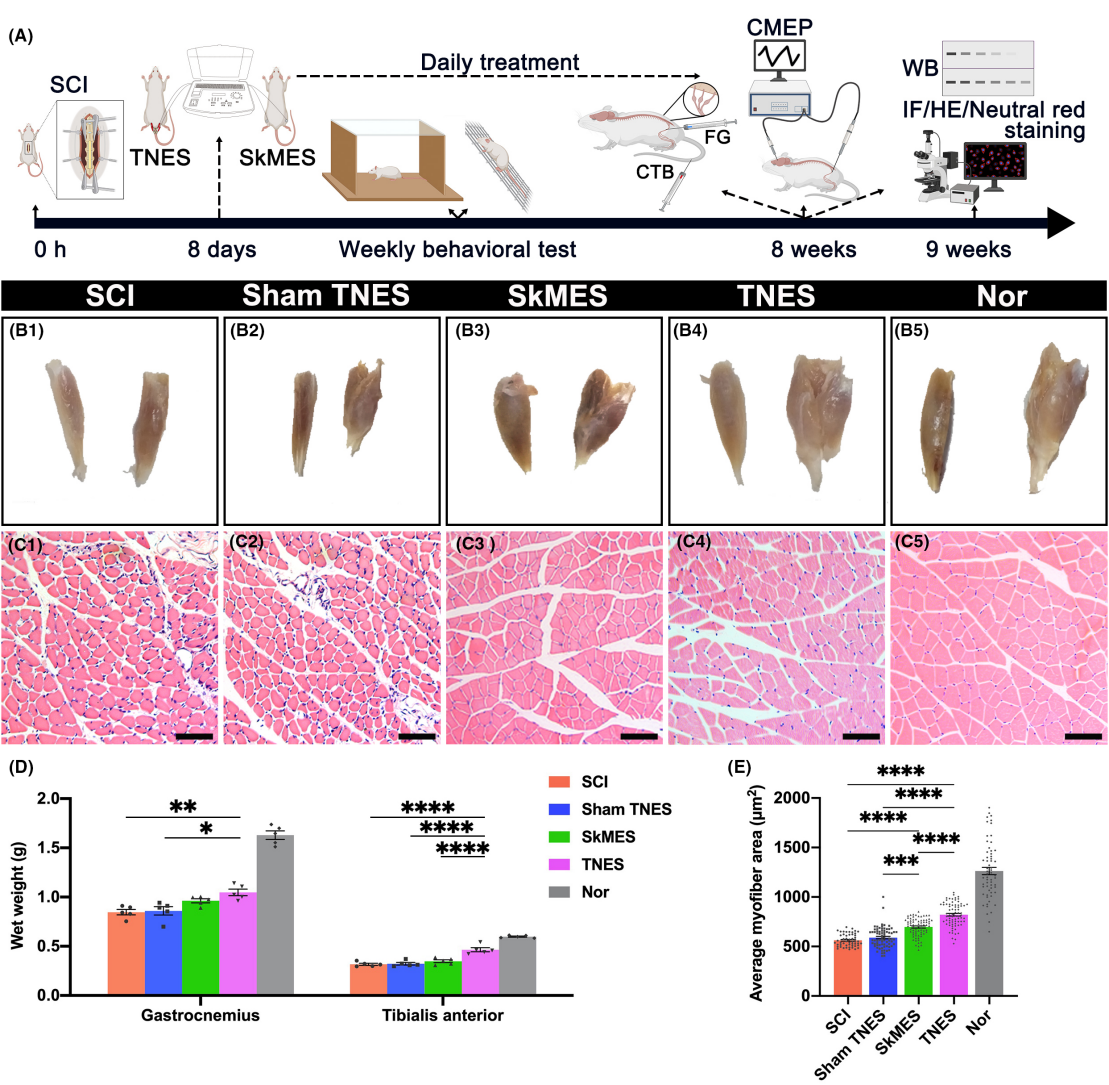
Tail nerve electrical stimulation (TNES) alleviated muscle atrophy in the hindlimbs. (A) Experimental paradigms illustrating the timelines of the major experimental manipulations. (B) The appearance of the tibialis anterior muscle (left) and the gastrocnemius muscle (right) in each group. TNES significantly alleviated atrophy in the tibialis anterior muscle and gastrocnemius muscle. (C) HE staining in transverse sections from the tibialis anterior muscle in each group. (D) Bar chart showing the wet weights of the gastrocnemius and tibialis anterior muscles (*n* = 5. Data are shown as mean ± SEM. One‐way anova followed by Bonferroni post‐hoc test was used, *p < 0.05, **p < 0.01, ****p < 0.0001). (E) Bar chart showing the mean myofiber areas (*n* = 5). Data are shown as mean ± SEM. One‐way anova followed by Bonferroni post‐hoc test was used, ***p < 0.001, ****p < 0.0001). Scale bar = 20 μm in (C1–C5).

Experimental paradigms illustrating the timelines of the major experimental manipulations are presented in Figure 2A. To evaluate the effect of TNES on hindlimb muscle atrophy, the wet weights and cross‐sectional area of myofibers of the tibialis anterior and gastrocnemius muscle were determined after 7 weeks of TNES treatment (Figure 2B–E). Severe atrophy was observed in the muscles of rats in the SCI and sham TNES groups. However, muscle atrophy in the SkMES group was alleviated to some extent and was significantly alleviated in the TNES group compared with the other three groups (Figure 2B1–B5,D). Next, we performed HE staining on tissue sections prepared from the tibialis anterior muscle (Figure 2C1–C5,E). We found that the cross‐sectional area of myofibers in the SCI and the sham TNES groups was significantly reduced while the extent of atrophy in the cross‐sectional area of myofibers in the SkMES group was reduced compared to that in the SCI and sham TNES groups. Although the cross‐sectional area of myofibers in the TNES group was significantly smaller than that in the normal group (Nor group), it was significantly higher than that in the other three groups. These results showed that TNES significantly alleviated muscle atrophy in the hindlimbs.

### 3.3 The survival of neurons in the lumbar spinal cord

**FIGURE 3:**
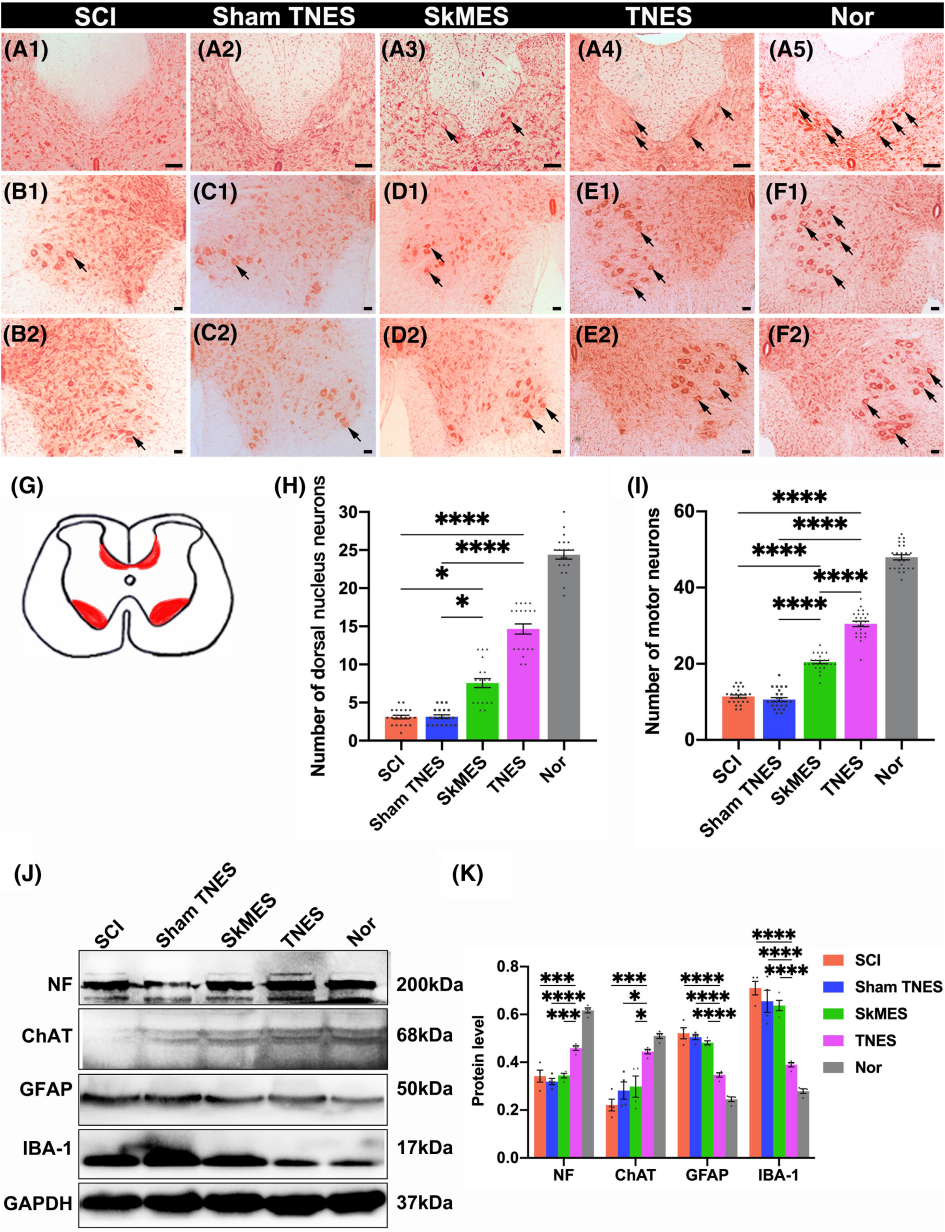
Tail nerve electrical stimulation (TNES) promoted the survival of spinal cord neurons. (A) Neutral red staining showing neurons in the L1 dorsal nucleus (arrows in A1–A5). The number of neurons in the L1 dorsal nucleus in the TNES group was higher than that in the SCI, sham TNES, and SkMES groups. (B–F) Neutral red staining showing motoneurons in the anterolateral horn of the L4 segment (arrows). The number of motoneurons in the TNES group (E1, E2) was higher than that in the SCI (B1, B2); sham TNES (C1, C2); and SkMES groups (D1, D2). (G) Sections of the spinal cord showing neurons in the dorsal nucleus and the anterolateral horn motoneurons. (H) Bar chart showing the number of neurons in the L1 dorsal nucleus (*n* = 5. Data are shown as mean ± SEM. Kruskal–Wallis test was used, *p < 0.05, ****p < 0.0001). (I) Bar chart showing the number of motoneurons in the anterolateral horn of the L4 segment (*n* = 5. Data are shown as mean ± SEM. One‐way anova followed by Bonferroni post‐hoc test was used, ****p < 0.0001). (J) Western blotting for NF, ChAT, GFAP, IBA‐1, and glyceraldehyde 3‐phosphate dehydrogenase (GAPDH) in the L1‐S2 segments of the spinal cord. The levels of NF and ChAT were higher and the levels of GFAP and IBA‐1 were lower in the TNES group than the SCI, sham TNES, and SkMES groups. (K) Bar chart showing the quantification of protein expression based on western blotting (*n* = 4. Data are shown as mean ± SEM. One‐way anova followed by Bonferroni post‐hoc test was used, *p < 0.05, ***p < 0.001, ****p < 0.0001). Scale bar = 100 μm in (A1–A5), 50 μm in (B1–F1, B2–F2).

Given that TNES alleviated hindlimb muscle atrophy through the lumbar spinal cord conduction pathway, we next considered the effect of this on the neurons. Sections from the L1 and L4 spinal cord were stained with neutral red, neurons in the L1 dorsal nucleus, and the L4 anterolateral horn motoneurons were included in the statistics (Figure 3A–I). We investigated neurons in the L1 dorsal nucleus and found that the number of neurons was reduced in the SCI group along with significant atrophy of the cell bodies. Changes in the sham TNES group were similar to those in the SCI group. However, the number of neurons in the L1 dorsal nucleus in the SkMES group was slightly higher than that in the SCI and sham TNES groups. Notably, the number of neurons in the L1 dorsal nucleus in the TNES group was significantly higher than that in the SCI and sham TNES groups while both the size and morphology of the neurons were similar to the Nor group (Figure 3A1–A5,G,H). Furthermore, we found that in the TNES group, the number of motoneurons in the anterolateral horn of the L4 segment that controls hindlimb movement was significantly greater than that in the SCI, sham TNES, and SkMES groups; the size of the cell bodies was closer to that of the Nor group (Figure 3B–F,G,I). In addition, the number of surviving motoneurons in the TNES group was consistent with the reduced degree of atrophy in the skeletal muscles. Western blotting results showed that the expression levels of neuronal proteins (marked by NF) and motor neuronal proteins (marked by choline acetyltransferase, ChAT) expressed in the spinal cord tissues of L1‐S2 segments in the TNES group were higher than those in the SCI, sham TNES, and SkMES groups (Figure 3J,K). However, these three groups also exhibited an increased number of astrocytes (marked by glial fibrillary acidic protein, GFAP) and microglial cells (marked by ionized Ca^2+^‐binding adapter protein 1, IBA‐1) (Figure 3J,K).

### 3.4 Tail nerve electrical stimulation increased the activation of the neural circuits in the lumbar spinal cord

**FIGURE 4:**
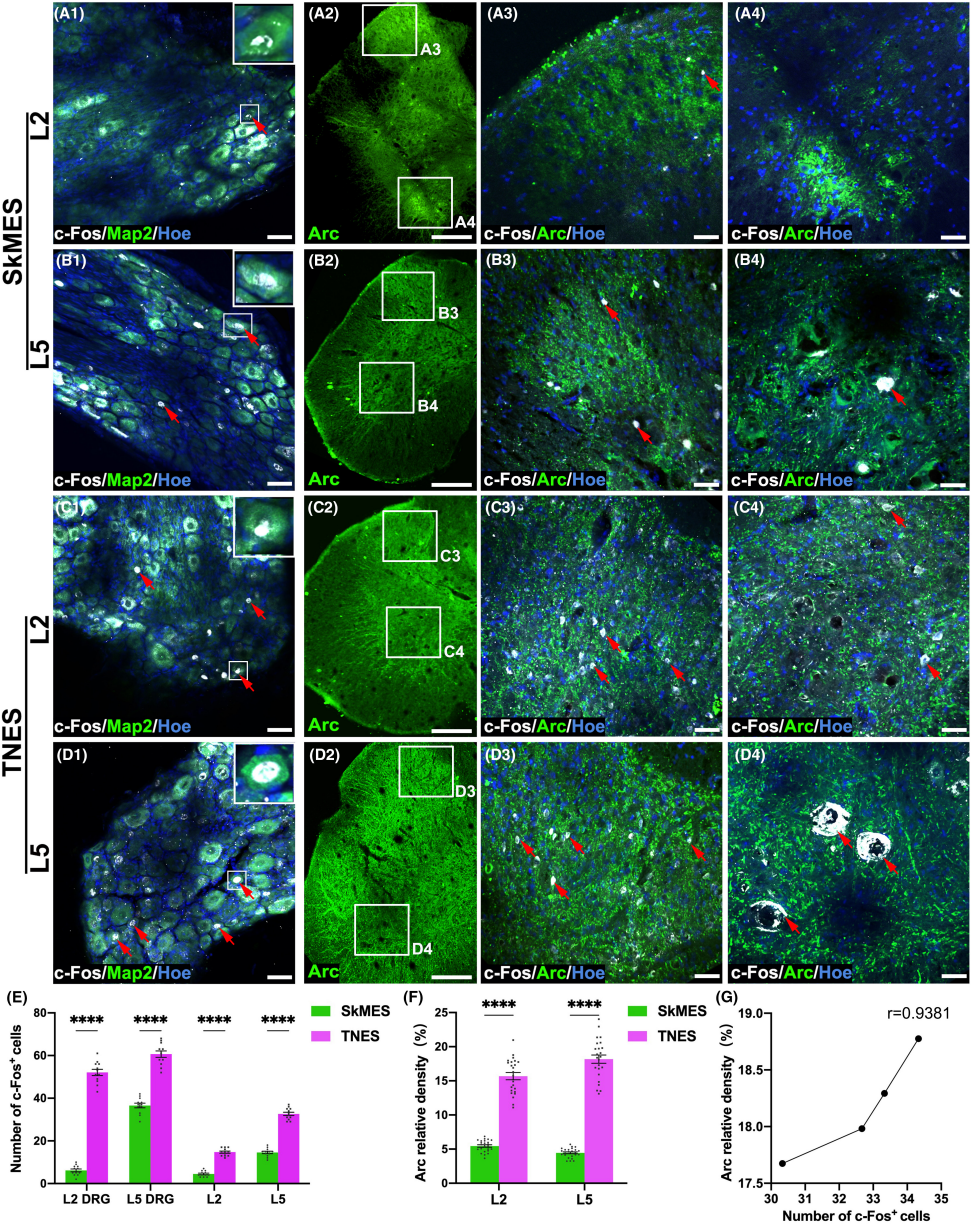
Tail nerve electrical stimulation (TNES) increased activation of the neural circuits in the lumbar spinal cord. In the SkMES group, the co‐expression of c‐Fos^+^ and microtubule‐associated protein 2 (Map2)^+^ is shown in the L2 (A1) and L5 (B1) DRG neurons (arrows). Inserts in the upper right corner of the images are the magnification of the area shown in the box; Arc^+^ neurons in the L2 (A2) and L5 (B2) segments is shown; the co‐expression of c‐Fos^+^ and Arc^+^ in the L2 (A3) and L5 (B3) dorsal horn neurons (arrows) is shown; the co‐expression of c‐Fos^+^ and Arc^+^ in the L2 (A4) and L5 (B4) ventral horn neurons is shown (arrows). In the TNES group, the co‐expression of c‐Fos^+^ and Map2^+^ in the L2 (C1) and L5 (D1) DRG neurons is shown (arrows). Inserts in the upper right corner of the images are the magnification of the area shown in the box; Arc^+^ neurons in the L2 (C2) and L5 (D2) segment is shown; the co‐expression of c‐Fos^+^ and Arc^+^ in the L2 (C3) and L5 (D3) dorsal horn neurons is shown (arrows); the co‐expression of c‐Fos^+^ and Arc^+^ in the L2 (C4) and L5 (D4) ventral horn neurons is shown (arrows). (E) Bar chart showing the number of c‐Fos^+^ neurons in the DRG, L2, and L5 spinal cord (*n* = 4. Data are shown as mean ± SEM. Unpaired *t* test was used, ****p < 0.0001). (F) Bar chart showing the relative density of Arc^+^ neurons in the L2 and L5 spinal cord (*n* = 4. Data are shown as mean ± SEM. Unpaired *t* test was used, ****p < 0.0001). (G) Line chart showing the linear relationship between the relative density of Arc^+^ and the number of c‐Fos^+^ neurons of L5 in the TNES group (*r* = 0.9381). Scale bar = 50 μm in (A1–D1), 200 μm in (A2–D2), and 50 μm in (A3–D3, A4–D4).

To further analyze whether the survival of neurons was related to the activation of the neural circuits and neural plasticity, we next focused on the expression of c‐Fos (immediate‐early gene product) and Arc, a sub‐immediate early gene related to neuroplasticity, in the dorsal root ganglions (DRGs), the L2 and L5 dorsal horn, and the ventral horn neurons in the SkMES and TNES groups at week 8 after electrical stimulation (Figure 4A–D). c‐Fos was predominantly expressed in the nuclei of neurons but also detected in the cytoplasm around the nucleus with large cell bodies. We detected a small number of c‐Fos^+^ neurons in the DRG of the L2 segment in the SkMES group, and the number of c‐Fos^+^ neurons was fewer than those in the DRG from the L5 segment after electrical stimulation. The expression of c‐Fos^+^ neurons in DRGs from the TNES group was significantly higher than that in the SkMES group, and the number of c‐Fos^+^ neurons in the DRG from the L5 segment was higher than that in the L2 segment (Figure 4A1–D1,E and Figure S1). Furthermore, the number of c‐Fos^+^ neurons in the L2 and L5 segments of the spinal cord in the SkMES group and the TNES group was consistent with that in the DRG. In the SkMES group, a small number of ventral horn motoneurons and dorsal horn neurons expressed c‐Fos in the L5 segment, while few c‐Fos^+^ neurons were observed in the L2 dorsal horn. In the TNES group, both the ventral horn motoneurons and dorsal horn neurons expressed c‐Fos in the L5 segment, while only the dorsal horn neurons expressed c‐Fos in the L2 segment; no c‐Fos^+^ motoneurons were detected in the ventral horn (Figure 4A2–D2,A3–D3,A4–D4,E). Arc protein was expressed in neuronal cell bodies and neuronal process terminals; therefore, we compared differences in the expression levels of Arc^+^ between the SkMES and TNES groups using fluorescence density statistics (Figure 4A2–D2,A3–D3,A4–D4). Our results analysis revealed that the expression levels of Arc in the L2 and L5 segments of the SkMES group were lower than those in the TNES group (Figure 4F); these findings were consistent with the expression of c‐Fos (Figure 4G). These results showed that TNES had a greater effect on the activation and plasticity regulation of the neural circuits in the lumbar spinal cord.

### 3.5 Tail nerve electrical stimulation regulated the plasticity of the motor neural circuits and restored the innervation of motoneurons

**FIGURE 5:**
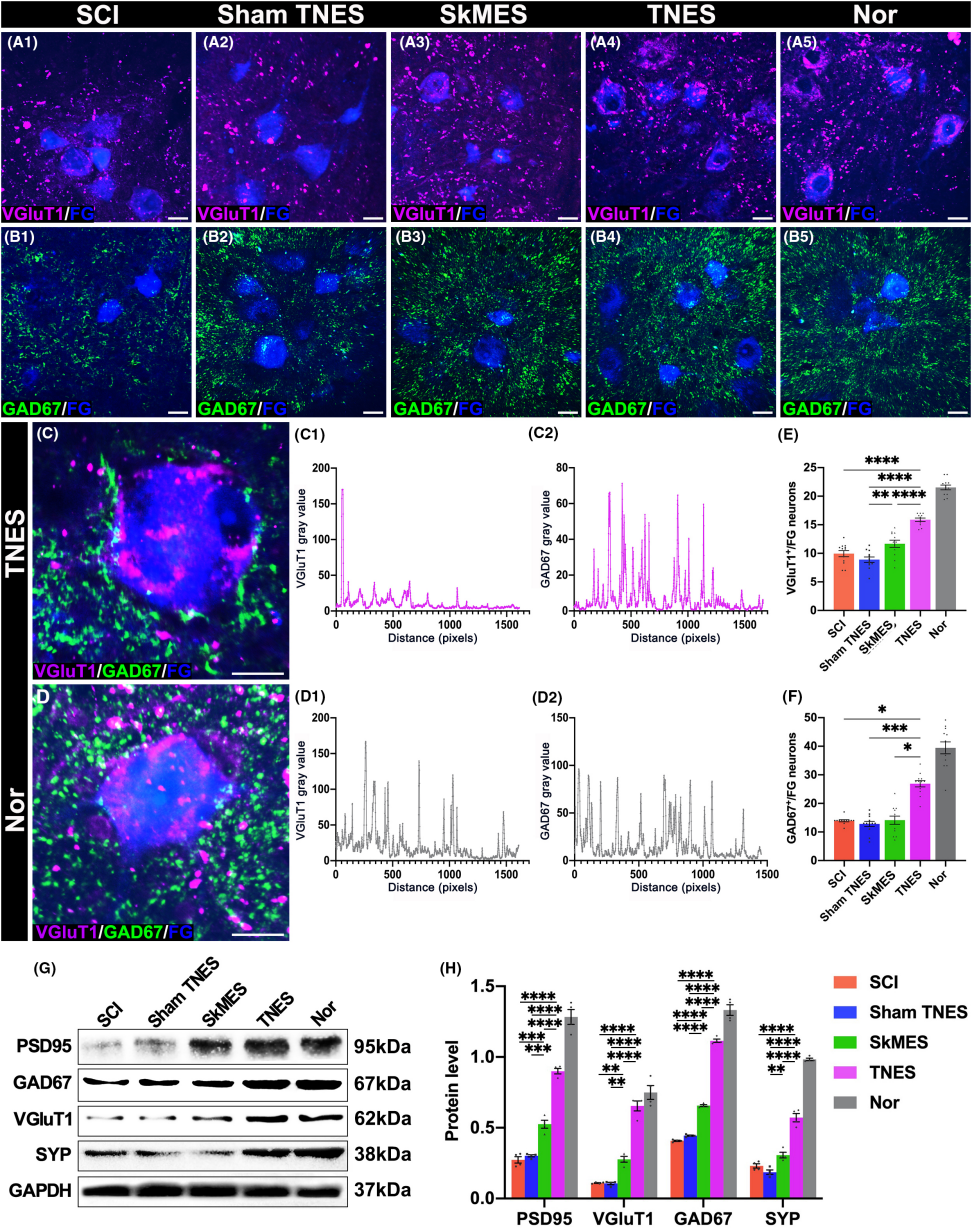
Tail nerve electrical stimulation (TNES) increased the number of presynaptic terminals on the surface of motoneurons. (A) VGluT1^+^ presynaptic terminals on the surface of FG^+^ motoneurons in each group. (B) GAD67^+^ presynaptic terminals on the surface of FG^+^ motoneurons in each group. (C) The co‐expression of VGluT1^+^ and GAD67^+^ presynaptic terminals in the TNES group. The chart shows gray value changes of VGluT1^+^ (C1) and GAD67^+^ (C2) presynaptic terminals on the surface of FG^+^ motoneurons in the TNES group. (D) The co‐expression of VGluT1^+^ and GAD67^+^ presynaptic terminals in the Nor group. The chart shows gray value changes of VGluT1^+^ (D1) and GAD67^+^ (D2) presynaptic terminals on the surface of FG^+^ motoneurons in the Nor group. Bar chart showing the gray value of presynaptic VGluT1^+^ (E) and GAD67^+^ (F) terminals on the surface of FG^+^ neurons in each group (*n* = 4. Data are shown as mean ± SEM. One‐way anova followed by Bonferroni post‐hoc test was used in (E), and Kruskal–Wallis test was used in (F), *p < 0.05, **p < 0.01, ***p < 0.001, ****p < 0.0001). (G) Western blotting in the L1‐S2 segments of the spinal cord. The levels of PSD95, GAD67, VGluT1, and SYP were higher in the TNES group than in the SCI, sham TNES, and SkMES groups. (H) Bar chart showing quantification of the protein expression based on western blots (*n* = 4. Data are shown as mean ± SEM. One‐way anova followed by Bonferroni's post‐hoc test was used, **p < 0.01, ***p < 0.001, ****p < 0.0001). Scale bar = 30 μm in (A1–A5, B1–B5), 20 μm in (C, D).

Having analyzed the expression levels of c‐Fos and Arc by investigating the role of TNES in regulating the plasticity of the motor neural circuits, we next used retrograde tracing with FG on the sciatic nerve. Then, we investigated the number of VGluT1^+^ and GAD67^+^ presynaptic terminals, representing excitatory and inhibitory innervation, respectively, on the surface of FG^+^ motoneurons which control hindlimb movement (Figure 5A–D). Our results showed that the numbers of VGluT1^+^ presynaptic terminals on the surface of FG^+^ neurons in the SCI and sham TNES groups were lower than those in the SkMES and TNES groups. Although the number of VGluT1^+^ presynaptic terminals in the TNES group was lower than that in the Nor group, it was significantly greater than that in the SkMES group (Figure 5E). These results showed that TNES activated the motor neural circuits and increased the amount of excitatory neural information received by motoneurons. The detection of GAD67 showed that the number of GAD67^+^ presynaptic terminals on the surface of FG^+^ motoneurons was significantly lower in the SCI, sham TNES, and SkMES groups. However, the number of GAD67^+^ presynaptic terminals in the TNES group was significantly greater than that in the other three groups (Figure 5F). Moreover, the results of western blotting showed that although the expression levels of postsynaptic density protein 95 (PSD95), SYP, VGluT1, and GAD67 in the L1‐S2 segments of the spinal cord in the TNES group were lower than those in the Nor group, these levels were higher than those in the other three groups (Figure 5G,H). These results revealed that activation of the motor neural circuits by TNES plays a positive role in maintaining the innervation of motoneurons.

### 3.6 Tail nerve electrical stimulation improved innervation and regeneration in muscles

**FIGURE 6:**
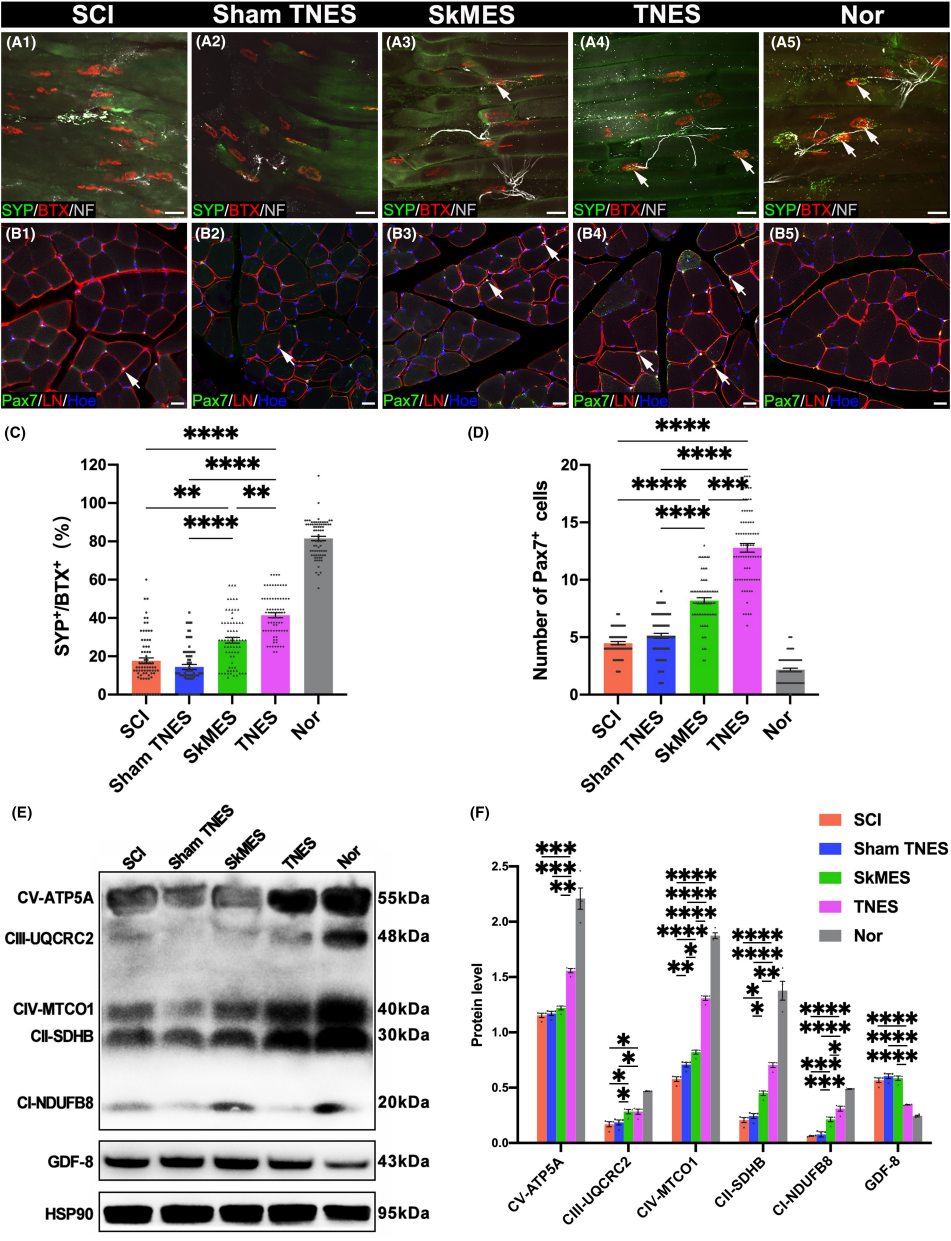
Tail nerve electrical stimulation (TNES) improved muscle innervation and regeneration. (A1–A5) BTX^+^ motor endplates with SYP^+^/NF^+^ nerve fibers in each group. (B1–B5) Representative images showing Pax7^+^ cells and LN in the extracellular matrix located between the tibialis anterior muscle. (C) Bar chart showing the ratio of SYP and BTX double‐positive motor endplates to the total BTX^+^ motor endplates (*n* = 5. Data are shown as mean ± SEM. Kruskal–Wallis test was used, **p < 0.01, ****p < 0.0001). (D) Bar chart showing the number of Pax7^+^ cells (*n* = 5. Data are shown as mean ± SEM. Kruskal–Wallis test was used, ***p < 0.001, ****p < 0.0001). (E) Western blot showing mitochondrial respiratory chain complex proteins and GAPDH in the gastrocnemius muscles. Compared with the SCI, sham TNES, and SkMES groups, the levels of mitochondrial respiratory chain complex proteins were increased in the TNES group; however, the levels of GDF‐8 were reduced in the TNES group. (F) Bar chart showing the quantification of protein expression based on western blots (*n* = 4. Data are shown as mean ± SEM. One‐way anova followed by Bonferroni post‐hoc test was used, *p < 0.05, **p < 0.01, ***p < 0.001, ****p < 0.0001). Scale bar = 50 μm in (A1–A5), 20 μm in (B1–B5).

Hindlimb muscle innervation and regeneration were detected (Figure 6A–D). We investigated the innervation of motor endplates in longitudinal sections prepared from the gastrocnemius muscles (Figure 6A1–A5 and Figure S2). Our results showed that α‐bungarotoxin positive (BTX)^+^ endplates were innervated by NF^+^ fibers and that the oval endplate surface was rich in the presynaptic protein SYP. In the SCI and sham TNES groups, the expression levels of SYP on the motor endplate surface had decreased significantly, and there was notable atrophy in NF^+^ fibers. The expression levels of SYP on the endplate surface in the SkMES group were higher than those in the SCI and sham TNES groups. However, the expression levels of SYP on the endplate surface were significantly increased in the TNES group (Figure 6C). These results showed that there was more abundant expression of SYP on the surface of endplates and the terminals of NF^+^ nerve fibers in the TNES group than the other groups. To investigate the effect of TNES on preventing muscle atrophy in the hindlimbs, we next focused on the activation of muscle satellite cells in transverse sections from the tibialis anterior muscles (Figure 6B1–B5 and Figure S3). Activated muscle satellite cells can divide and proliferate and are known to express Pax7. Our results showed that few Pax7^+^ cells were detected in normal muscles although higher numbers were detected in denervated muscle.

Following direct muscle electrical stimulation in the SkMES group (electrodes were placed on the muscle belly of the tibialis anterior and gastrocnemius muscles of the hindlimbs), the number of Pax7^+^ cells in the SkMES group was significantly higher than that in the SCI and sham TNES groups. However, the number of Pax7^+^ cells in the TNES group was significantly higher than that in the SkMES group (Figure 6D). Furthermore, western blotting using samples of gastrocnemius muscles showed that the levels of mitochondrial respiratory chain complex protein were significantly lower in the SCI group than in the Nor group. However, the TNES group expressed more mitochondrial respiratory chain complex protein than the SCI, sham TNES, and SkMES groups (Figure 6E,F). These results suggested that TNES restored muscle innervation while promoting muscle regeneration and energy metabolism. In addition, our analysis showed that the expression levels of growth differentiation factor 8 (GDF8) protein in the gastrocnemius muscle of the TNES group were significantly lower than those in the SCI, sham TNES, and SkMES groups (Figure 6E,F). Collectively, these results showed that TNES effectively repaired the inhibitory effect of denervation on muscle regeneration after SCI.

### 3.7 Tail nerve electrical stimulation improved the hindlimb movement, electrophysiological function, and nerve regeneration

**FIGURE 7:**
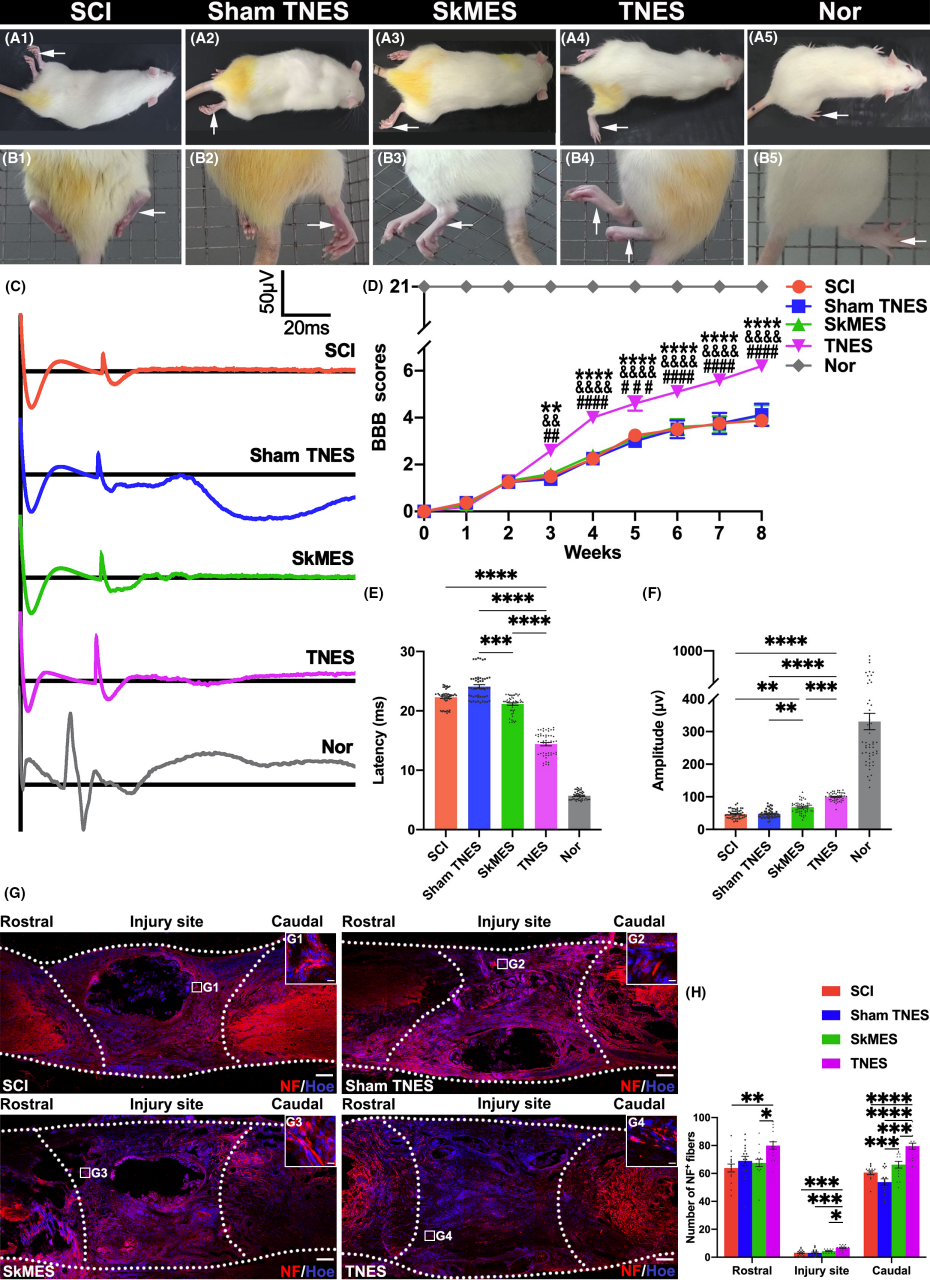
Tail nerve electrical stimulation (TNES) improved hindlimb movement, electrophysiological function, and nerve regeneration. (A1–A5) BBB assessment was performed in each group. (B1–B5) The grid climbing test was performed. (C) CMEPs were obtained by electrophysiological analysis. (D) Comparison of BBB scores for hindlimb locomotor function (*n* = 8 in SCI, sham TNES, and Nor group; *n* = 10 in the SkMES and TNES groups; ^#, &,^ * indicate statistical significance compared with the SCI, sham TNES, and SkMES groups, respectively) ^##^ *p* < 0.01; ^&&^ *p* < 0.01; **p < 0.01; ^###^ *p* < 0.001; ^####^ *p* < 0.0001; ^&&&&^ *p* < 0.0001; ****p < 0.0001. (E) Bar charts of CMEPs latency showing a shorter latency in the TNES group than the SCI, sham TNES, and SkMES groups (*n* = 5. Data are shown as mean ± SEM. Kruskal–Wallis test was used, ***p < 0.001, ****p < 0.0001). (F) Bar charts of CMEPs amplitude, showing higher amplitudes in the TNES group than the SCI and sham TNES groups (*n* = 5. Data are shown as mean ± SEM. Kruskal–Wallis test was used, **p < 0.01, ****p < 0.0001). (G) NF^+^ nerve fibers at the site of SCI and its rostral and caudal sites. (G1–G4) The pictures are the magnification of the area indicated in the box area of (G). The dotted lines delineate the contours of longitudinal sectioning of the spinal cord and the scope of the injury area corresponding to the injury length of 2 mm. (H) Bar chart showing the number of NF^+^ nerve fibers in the regions rostral and caudal to/in the injury site (*n* = 5). Data are shown as mean ± SEM. Kruskal–Wallis test was used in the rostral regions and injury site and one‐way anova followed by Bonferroni's post‐hoc test was used in the caudal regions, *p < 0.05, **p < 0.01, ***p < 0.001, ****p < 0.0001). Scale bar = 200 μm in (G), 10 μm in (G1‐G4).

The BBB test indicated further improvement in voluntary locomotor function, as evidenced by the frequent movement of large joints and the achievement of coordinated forelimb and hindlimb movement in rats receiving TNES treatment (Figure 7A1–A5). Furthermore, the hindlimbs exhibited coordinated left and right pedaling on the grid in the TNES group in the grid climbing test (Figure 7B1–B5). The BBB scores for the three groups were significantly lower than those in the TNES group. In addition, CMEPs showed that rats in the TNES group exhibited a higher amplitude and shorter latency; these were significantly different from those in the SCI, sham TNES, and SkMES groups (Figure 7C–F). Collectively, these results showed that TNES increased the ability of the lumbar spinal cord to respond to motor control. NF immunofluorescence was also performed on the rats (Figure 7G and Figure S4). At the caudal position of the injury, the number of NF^+^ nerve fibers in the SkMES group was significantly higher than that in the sham TNES group; the TNES group had the largest number of NF^+^ nerve fibers. The number of NF^+^ nerve fibers in the lesion center in the TNES group was significantly higher than that in the other groups. At the rostral position of the injury, there was no statistically significant difference in the number of NF^+^ nerve fibers compared with the SkMES, sham TNES, and SCI groups. The number of NF^+^ nerve fibers in the TNES group was significantly greater than that in the SkMES and SCI groups (Figure 7H).

### 3.8 Neural tracing revealed the pathway by which TNES activated and regulated plasticity of the motor neural circuits in the lumbar spinal cord

**FIGURE 8:**
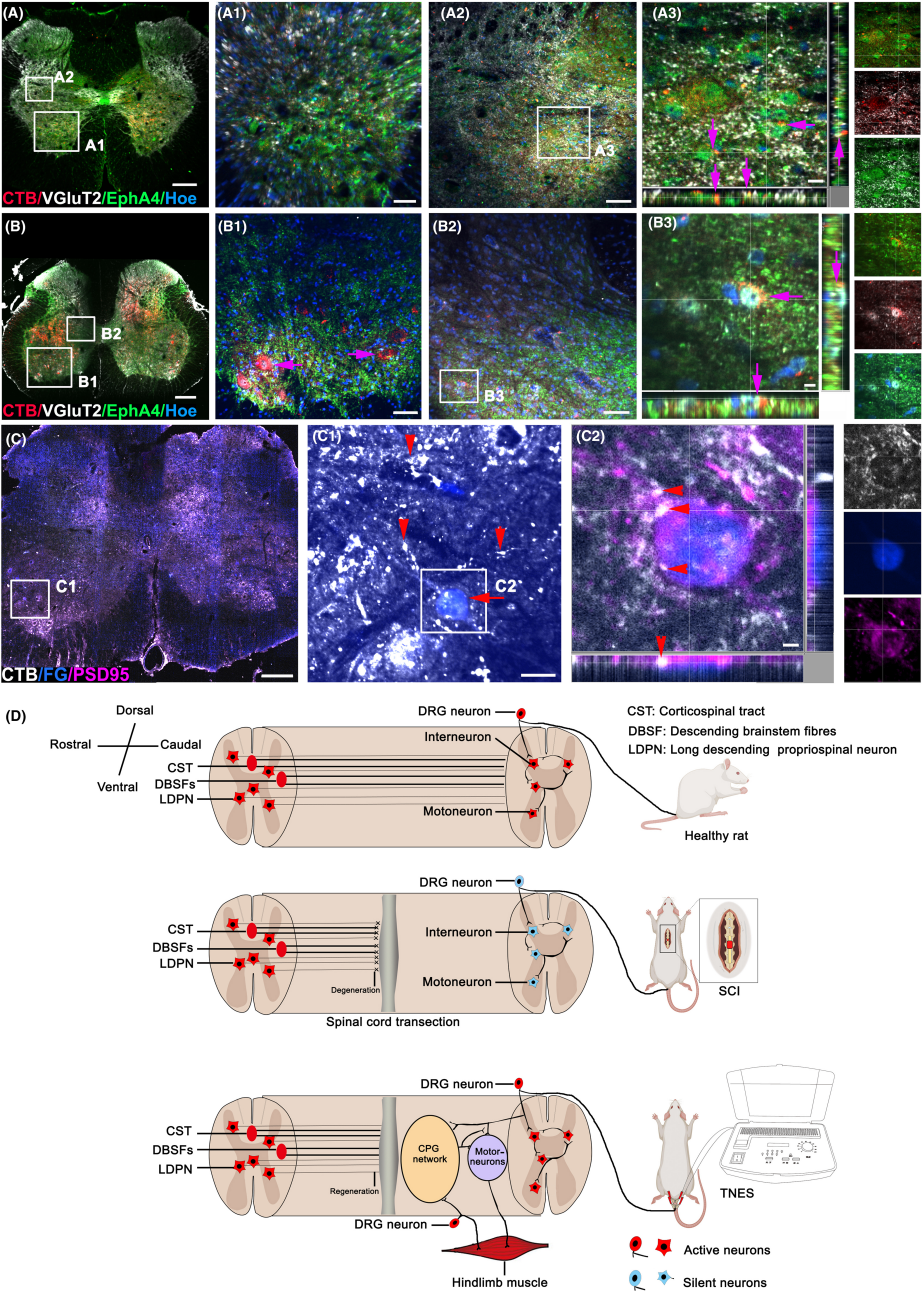
Retrograde nerve tracing demonstrated the specific neural pathway of tail nerve electrical stimulation (TNES). (A) Representative images showing CTB^+^ afferent nerve fibers in the L2 segment of the TNES group. (A1) Magnified image in the boxed area of (A) showing CTB^+^ afferent nerve fibers in the L2 ventral horn. (A2) Magnified image in the boxed area of (A) showing CTB^+^ afferent nerve fibers contacting VgluT2^+^/EPHA4^+^ CPG neurons in the middle lamellae of the L2 gray matter (arrows in A3). (A3) Magnified image in the boxed area of (A2). (B) Representative images showing CTB^+^ afferent nerve fibers and neurons in the L5 segment of the TNES group. (B1) Magnified image in the boxed area of (B) showing CTB^+^ afferent nerve fibers and motoneurons in the L5 ventral horn. (B2) Magnified image in the boxed area of (B) showing CTB^+^ afferent nerve fibers contacting VgluT2^+^/EPHA4^+^ CPG neurons in the middle lamellae of the L5 gray matter (arrows in B3). (B3) Magnified image in the boxed area of (B2). (C) Representative images showing FG^+^ motoneurons and CTB^+^ nerve fibers and neurons in the L5 segment of the TNES group. (C1) Magnified image in the boxed area of (C) showing that CTB^+^ nerve fibers (arrowheads) and FG^+^ motoneuron cell bodies (arrow) formed abundant close contacts with the CTB^+^ presynaptic terminals in the L5 ventral horn. (C2) Magnified image in the boxed area of (C1) showing the co‐expression of FG^+^ motoneurons, CTB^+^ presynaptic terminals and PSD95 protein (arrowheads). (D) A schematic diagram showing the specific neural pathway and the mechanism of activation in the spinal cord CPG and effects on the motor neural circuits during TNES treatment. Scale bar = 300 μm in (A–C), 70 μm in (A1–B1, A2–B2), 20 μm in (A3), 10 μm in (B3, C2), and 30 μm in (C1).

To determine the specific neural pathway of TNES acting on the lumbar spinal cord to restore motor neuron and muscle innervation, we performed retrograde CTB tracing in the tail nerve. In the L2 segment, we observed that the CTB^+^ central branch presynaptic terminals of DRG neurons formed close contacts with VgluT2^+^/EphA4^+^ interneurons in the middle lamina of the spinal gray matter (Figure 8A,A1–A3). In the L5 segment, CTB was successfully delivered to motoneurons (Figure 8B,B1). We also observed CTB in the central branch presynaptic terminals of DRG neurons, which formed close contacts with vesicular glutamate transporters 2 (VgluT2)^+^/ephrin A4 receptor (EphA4) ^+^ interneurons (Figure 8B2,B3). Then, we performed CTB tracing on the tail nerve and FG tracing on the sciatic nerve; analysis showed that in the L5 segment, the FG^+^ motor neuron cell bodies innervating the hindlimb muscles formed abundant close contacts with the CTB^+^ presynaptic terminals. Furthermore, the cell bodies of FG^+^ neurons and CTB^+^ motoneurons were both detected at the transverse level of the L5 segment (Figure 8C,C1,C2). Collectively, these results showed that the neural information provided by TNES could affect the motor neural circuits of the lumbar spinal cord via the afferent effects of the DRG and then affected the VGluT2^+^/EphA4^+^ CPG neurons and the motoneurons innervating the hindlimb muscles (Figure 8D).

## 4 DISCUSSION

The main factors that make it difficult to recover locomotor function after thoracic spinal cord transection injury include neuronal atrophy and apoptosis after the loss of brain innervation in the lumbar spinal cord and irreversible muscular atrophy in the paralyzed hindlimbs.^5^, ^6^ Preventing neuronal and muscle atrophy and apoptosis during the early stages of injury is of great significance for the reconstruction of voluntary locomotor function.^24^, ^25^

We performed TNES treatment 1 week after T10 spinal cord transection injury. Our analysis showed that TNES played an important role in activating the motor neural circuits of the lumbosacral spinal cord, thereby preventing a reduction in the number of neurons and regulating the plasticity of the neural circuits and preventing the atrophy of hindlimb muscles. Owing to the non‐invasive nature and safety of TNES, the mechanisms of its therapeutic effect could provide an important theoretical reference for the development of lumbosacral spinal cord transcutaneous electrical stimulation therapy for SCI repair in the future.

### 4.1 Tail nerve electrical stimulation prevented skeletal muscle atrophy via spinal cord conduction pathways

The basic cause of skeletal muscle atrophy after SCI is the loss of excitatory afferent to motoneurons; this leads to the loss of motor innervation in the skeletal muscle.^6^, ^24^ In recent years, many studies have confirmed that spinal cord EES or ISMS could induce skeletal muscle contraction and limb movement.^11^, ^12^, ^18^ However, there is still a lack of research relating to the specific mechanisms underlying spinal nerve stimulation in preventing neuronal apoptosis and muscular atrophy. Studies by Zhang et al and our own research group have shown that TNES can also cause contraction of the muscles in the hindlimbs.^6^, ^19^ However, these previous studies did not specifically analyze the neural conduction pathways and mechanisms underlying the effects of TNES on the prevention of skeletal muscle atrophy.

In the present study, we performed TNES and found that the electrical signals triggered by TNES could not be recorded in the sensorimotor cortex or forelimb muscles through the transected spinal cord but could be recorded in the muscles of the bilateral hindlimbs. However, we failed to record electrical signals in the muscles of the hindlimbs when we performed TNES after blocking the tail nerve with lidocaine. These results suggested that the promoting effect of TNES on the regeneration of brain‐derived nerve fibers (such as CST and rubrospinal tract) might be limited, but the electrical signals provided by TNES could be transmitted to the spinal cord via the sacrococcygeal afferent nerve fibers and then activate the motor neural circuits of the spinal cord, thereby causing contraction of the hindlimb muscles. This is most likely why TNES treatment effectively prevented atrophy of the hindlimb muscles. However, further research should investigate the biological effects associated with the activation of the lumbar spinal cord neural circuits.

### 4.2 Tail nerve electrical stimulation prevented neuron loss in the spinal cord and reconstructed the motor neural circuits

After 7 weeks of TNES treatment, the number of neurons in the lumbar spinal cord were significantly greater than those in rats receiving stimulation in the hindlimb muscles (SkMES group). We hypothesized that TNES could effectively activate DRG neurons and then activate sensory neurons in the dorsal nucleus and finally activate interneurons and motoneurons in the ventral horn. The electrical signals provided by TNES likely play an important role in maintaining neuronal excitability and preventing neuronal apoptosis. Many studies have reported that central nervous stimulation plays an important role in activating and reconstructing the neural circuits.^10^, ^12^, ^26^ In this study, we found that the expression levels of the neuronal marker NF and the motor neuron marker ChAT increased significantly in the lumbar spinal cord, while expression levels of GFAP and IBA‐1 decreased significantly. These results suggest that the survival of neurons increased after TNES treatment, while the activation of astrocytes and microglia caused by neuronal apoptosis decreased.^27^

The detection of c‐Fos (expressed in neuronal activation) and Arc (synaptic plasticity‐related protein) further confirmed the hypothesis that TNES activated DRG neurons and the spinal neurons and reconstructed the motor neural circuits.^28^ c‐Fos and Arc were barely detectable in DRG neurons and spinal cord neurons in the SCI and sham TNES groups. By contrast, a small number of DRG and spinal cord neurons expressing c‐Fos and Arc were detected in the L2 and L5 spinal cord segments of rats receiving stimulation in the hindlimb muscles (SkMES group), although the numbers of these neurons were significantly smaller than those in the TNES group. Collectively, these results suggested that TNES activated and regulated the plasticity of the spinal motor neural circuits more effectively than SkMES. These results also revealed a high correlation between c‐Fos and Arc expression, thus suggesting that synaptic plasticity changes in the neurons after activation, especially motoneurons, and may be an important reason for the improvement in skeletal muscle atrophy. Courtine et al. found that EES of the lumbosacral DRG is more effective than stimulation of the dorsal column of the spinal cord in terms of activating motoneurons to produce movement in the paralyzed limb.^11^ Interestingly, TNES exerts effects that are similar to EES on the activation of DRGs and the motor neural circuits and therefore has important clinical application value.

### 4.3 Tail nerve electrical stimulation restored skeletal muscle innervation and skeletal muscle function

Previous studies on spinal cord stimulation focused more on the immediate functional effects and less on the effects of long‐term regular cumulative stimulation on synaptic plasticity.^4^, ^12^, ^29^ Clinical rehabilitation by nerve stimulation focused more on the observation of rehabilitation effects.^30^, ^31^, ^32^ However, it was difficult to analyze the structural basis of plasticity regulation of the neural circuits involved in neural rehabilitation at the cellular level. We focused on motoneurons in the ventral horn of the spinal cord that innervate hindlimb movement and found that there were abundant excitatory and inhibitory presynaptic terminals on the surface of motoneurons in the Nor group. However, the numbers of these two types of presynaptic terminals on the surface of motoneurons were significantly decreased in SCI rats. Remarkably, electrical stimulation of the hindlimb muscle (SkMES group) could increase the number of excitatory presynaptic terminals on the surface of motoneurons to a certain extent. Although the number of presynaptic terminals on the surface of motoneurons in the TNES group was still lower than that in the Nor group, it was significantly higher than in the other groups. These results suggested that TNES could more effectively reconstruct the motor neural circuits and regulate the afferent information of motoneurons than electrical stimulation of the hindlimb muscle (SkMES group).

The expression levels of the synaptic marker SYP on the surface of the muscle motor endplates were higher in the TNES group than in the other groups. In addition, a greater number of Pax7^+^ muscle stem cells were also found to be activated by TNES. This suggested that the activation of motoneurons also affected the skeletal muscle, which further promoted the innervation and regeneration of skeletal muscle. These regenerated skeletal muscles expressed higher levels of mitochondrial respiratory chain protein complexes, thus indicating higher levels of energy metabolism and better functionality.^33^ Furthermore, several studies have confirmed that the recovery of skeletal muscle innervation is beneficial for the muscles to recruit neurotrophin and achieve regeneration.^34^, ^35^, ^36^ Subsequently, the muscle fibers also secreted myogenic trophic factors to influence the survival and function of motoneurons in a retrograde manner.^37^ In addition, some studies have reported that electrical stimulation of the skeletal muscle in situ can prevent atrophy; this might be because of improvements of muscle circulation and metabolism.^24^, ^30^, ^38^ However, our results suggest that electrical stimulation of the skeletal muscle in situ could not activate the spinal motor neural circuits in the SkMES group as effectively as in the TNES group. Therefore, SkMES was less efficient with regard to preventing skeletal muscle denervation and atrophy.

### 4.4 Neural circuit tracing and mechanism analysis of TNES

Behavioral tests confirmed that TNES effectively activated the motor neural circuits and promoted reconstruction of locomotor function. Furthermore, CMEPs exhibited higher amplitudes and shorter latencies, thus suggesting that motoneurons in the TNES group could respond more efficiently to motor information arising from the motor cortex. We speculate that TNES may reduce the excitation threshold of neurons in the lumbosacral spinal cord, which causes these neurons more likely to be excited by the motor neural information of the brain and generate action potentials.

Retrograde CTB tracing of the tail nerve confirmed the direction of nerve conduction of TNES through sensory afferent pathways and then to CPG neurons and motoneurons in the lumbar spinal cord. The results of double retrograde tracing of CTB in the tail nerve and FG in the sciatic nerve also confirmed that the stimulation signal in the tail nerve could directly affect the motoneurons innervating hindlimb movement at the level of the L5 spinal cord. After receiving TNES, the motor nerve fibers in the rat tail can generate nerve impulses that are transmitted to the motor neurons of lumbar spinal cord. This retrograde electrical signal is able to cause the postsynaptic membrane depolarization of motor neurons, resulting in calcium influx to increase the level of intracellular calcium ions: on the one hand, through the regulation of calcium/calmodulin‐dependent protein kinase signaling pathway, the synthesis and secretion of neurotrophic factors are increased, in which the axon terminals of neighboring neurons are attracted to form synaptic connections with the motor neurons,^39^, ^40^ and on the other hand, it may enhance the excitability of the motor neurons and maintain the structure and function of the motor endplates of the innervating muscles.^41^, ^42^ Collectively, these results confirm that TNES has a specific nerve pathway that efficiently activates the spinal motor neural circuits, restores the innervation of skeletal muscles in the hindlimbs, and prevents muscle atrophy. According to existing preclinical and clinical spinal cord stimulation studies, it could be speculated that primates and humans have neural circuits with a similar structural basis and share similar biological functional responses to sacrococcygeal nerve stimulation.^12^, ^43^ TNES functions by effectively activating the CPG neural circuit of the lumbar spinal cord through afferent information from the TNES, thereby activating the neurons that control the movement of the hindlimbs and maintaining skeletal muscle excitability. This prevents skeletal muscle atrophy. By contrast, the stimulation site of SkMES is at the skeletal muscle, which is conducive to increasing the blood circulation of skeletal muscle and maintaining a certain degree of excitability of the skeletal muscle.^37^ The results suggested that electrical stimulation of the hindlimb muscles (SkMES group) had a lower effect than TNES on preventing skeletal muscle atrophy and preventing the atrophy and number reduction of L1 dorsal nucleus neurons and L4 motor neurons, but a significantly higher effect than the SCI and Sham TNES groups. c‐Fos detection suggested that SkMES could also activate the spinal cord neural circuit to a certain extent, but the efficiency was lower than that of TNES. It may be because the distribution of sensory nerve fibers in the tibialis anterior and gastrocnemius muscles is more dispersed than that of sensory afferent nerve fibers in the tail nerve, which makes SkMES less efficient than TNES in activating spinal cord neural circuits. Further research will be needed to identify more effective SkMES therapy parameters to activate spinal neural circuits (such as increasing or decreasing the frequency of stimulation and extending the duration of each stimulation treatment) within the range of animal tolerance.

## 5 CONCLUSION

We confirmed that TNES could effectively activate the CPG neural circuits below the injury site of the spinal cord, regulate the synaptic plasticity of the motor neural circuits, and restore innervation of the skeletal muscle. TNES can therefore play a positive role in the prevention of muscular atrophy and the repair of locomotor function in rats with complete SCI. TNES is safer than other invasive spinal cord stimulation techniques and can readily be translated into clinical practice. Thus, it can be said that if timely and effective intervention measures cannot be provided after acute complete SCI, lumbosacral nerve electrical stimulation should be given in advance to maintain the excitatory function of the motor nerve circuit in the spinal cord below the injury area and prevent obvious atrophy of hindlimb muscles. We believe that our study has laid a structural and functional foundation for the future combination use of cutting‐edge biological treatment strategies to restore the voluntary movement of paralyzed hindlimbs.

## AUTHOR CONTRIBUTIONS

Bi‐Qin Lai, Ying Ding, and Yuan‐Shan Zeng designed and supervised the study. Jia‐Lin Liu, Zheng‐Hong Chen, Rong‐Jie Wu, Hai‐Yang Yu, Shang‐Bin Yang, Jing Xu, Chuang‐Ran Wu, Yi‐Nan Guo, Nan Hua, Xiang Zeng, Yuan‐Huan Ma, Ge Li, Ling Zhang, and Yuan‐Feng Chen performed the experiments and collected the data. Jia‐Lin Liu, Zheng‐Hong Chen, and Bi‐Qin Lai summarized, analyzed, and plotted the data and drafted the manuscript. Xiang Zeng, Yuan‐Huan Ma, Ge Li, Ling Zhang, Yuan‐Feng Chen, Yuan‐Shan Zeng, and Ying Ding helped with study planning and critically reviewed the manuscript. Jia‐Lin Liu, Bi‐Qin Lai, Yuan‐Shan Zeng, and Ying Ding wrote and finalized the article.

## CONFLICT OF INTEREST STATEMENT

The authors declare that they have no conflict of interest. Animals were used with the approval of the ethics committee of Sun Yat‐sen University (Animal Use Protocol no. SYSU‐IACUC‐2019‐B1101).

## Supporting information


Video S1.

Video S2.

Video S3.

Video S4.

Video S5.

Video S6.

Video S7.

Video S8.

Video S9.

Video S10.

Video S11.

Video S12.

Figure S1.

Figure S2.

Figure S3.

Figure S4.

Table S1.

Data S1.

Data S2.

## Data Availability

The data that support the findings of this study are available from the corresponding author upon reasonable request. The data that support the findings of this study are available in the supplementary material of this article.
