# Supplementary figures and images for: Effects of tail nerve electrical stimulation on the activation and plasticity of the lumbar locomotor circuits and the prevention of skeletal muscle atrophy after spinal cord transection in rats
Source: CNS Neurosci Ther. 2023 Sep 26;30(3):e14445. doi: 10.1111/cns.14445 (PMC10916423; doi:10.1111/cns.14445)

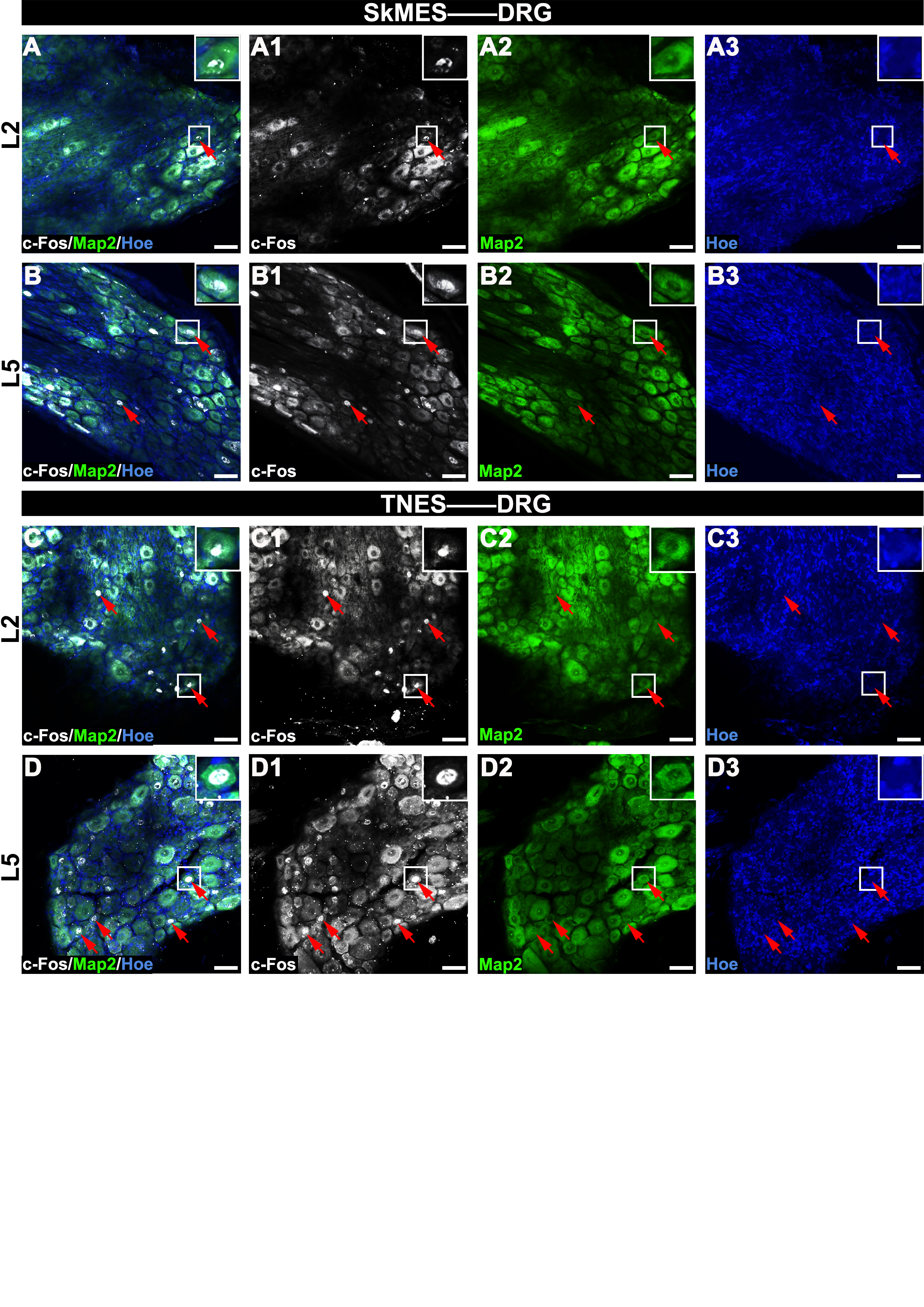

Supplement: Supplementary file 13 — Figure S1. [file CNS-30-e14445-s008.jpg]

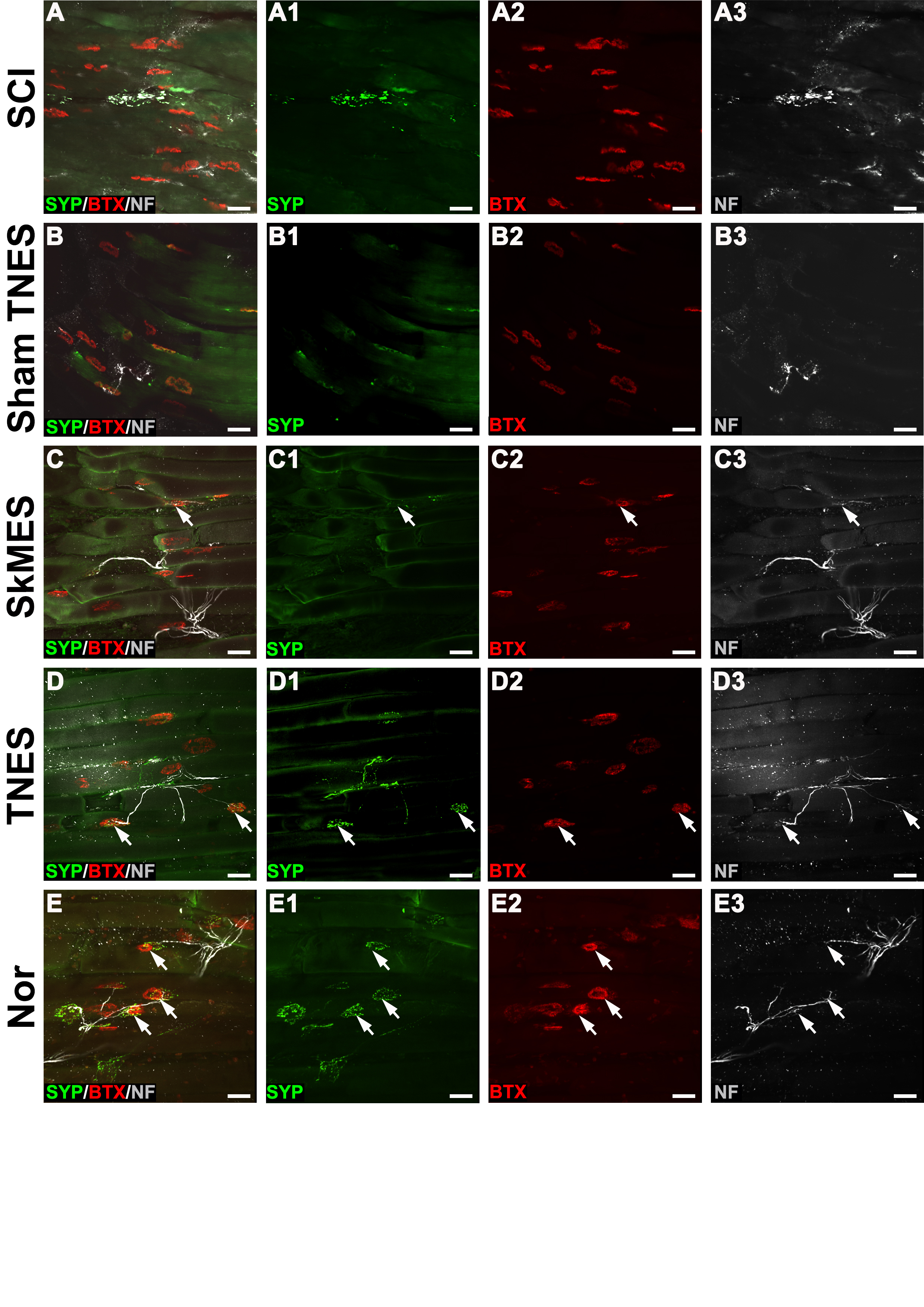

Supplement: Supplementary file 14 — Figure S2. [file CNS-30-e14445-s007.jpg]

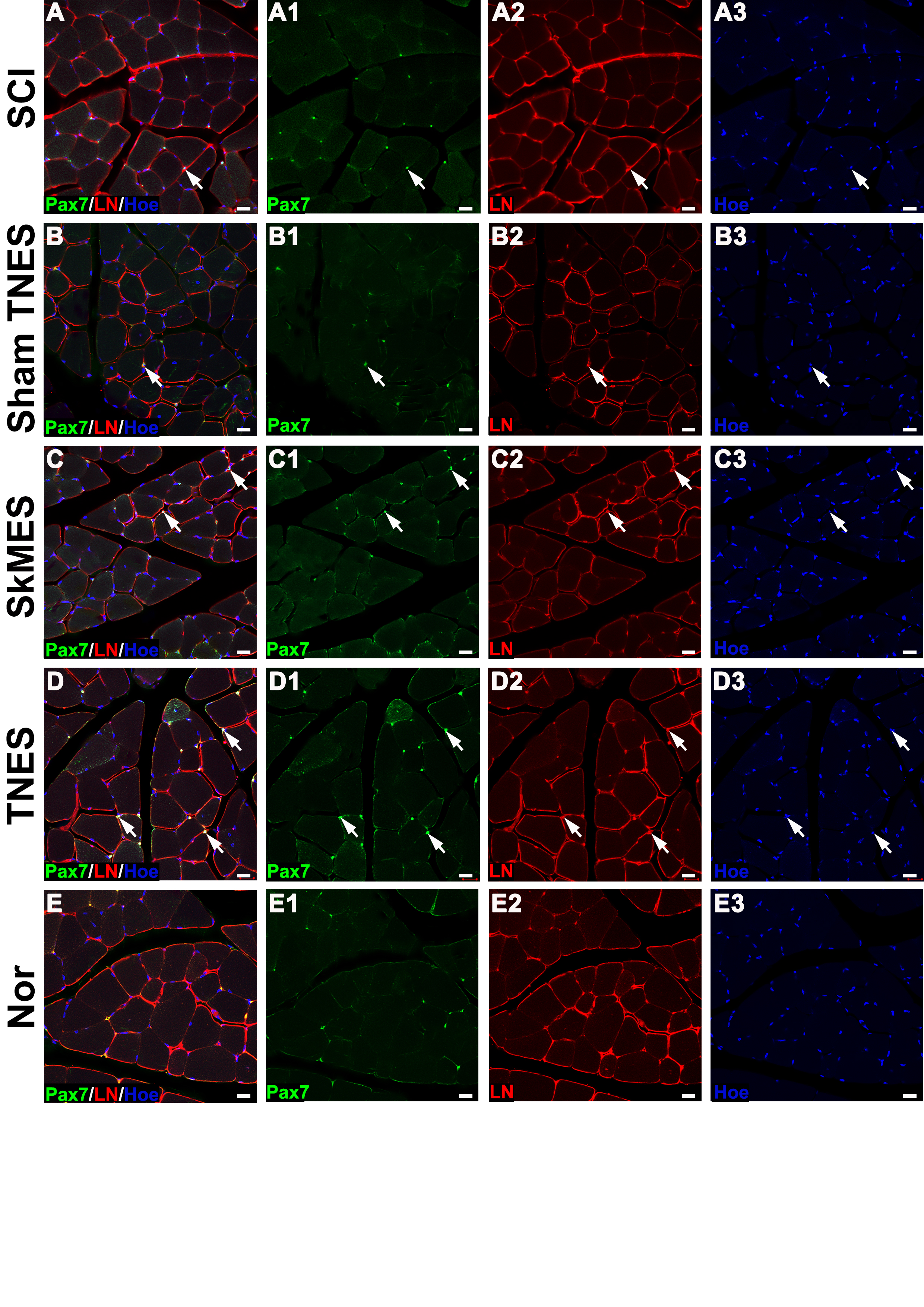

Supplement: Supplementary file 15 — Figure S3. [file CNS-30-e14445-s001.jpg]

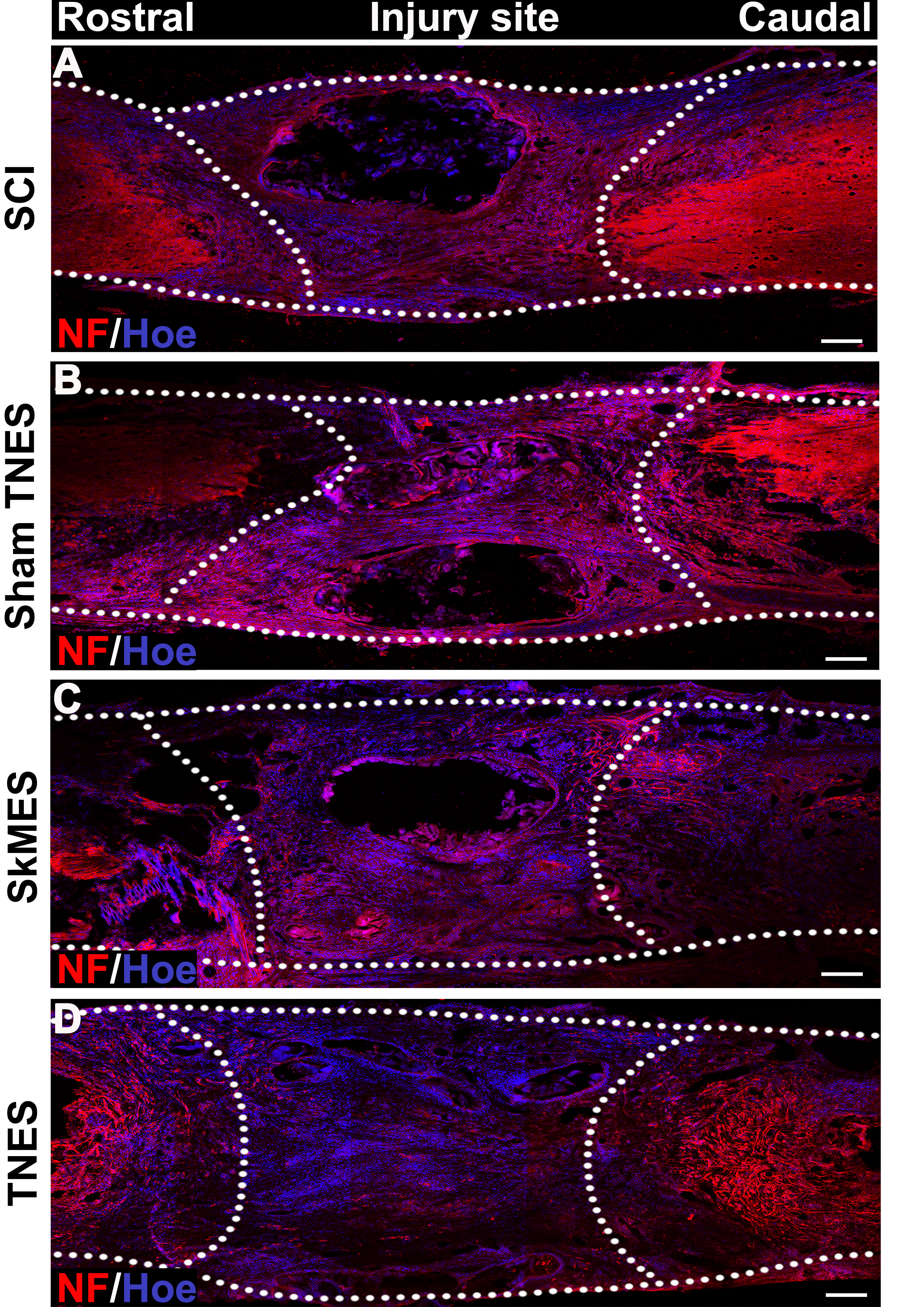

Supplement: Supplementary file 16 — Figure S4. [file CNS-30-e14445-s015.jpg]
