# Supplementary material for: Effects of tail nerve electrical stimulation on the activation and plasticity of the lumbar locomotor circuits and the prevention of skeletal muscle atrophy after spinal cord transection in rats
Source: CNS Neurosci Ther. 2023 Sep 26;30(3):e14445. doi: 10.1111/cns.14445 (PMC10916423; doi:10.1111/cns.14445)
Supplement: Supplementary file 17 — Table S1. [file CNS-30-e14445-s011.docx]

**Sup. TABLE. Primary and secondary antibodies**

| Antibodies | Species | Type | Dilution | Source (Catalog) |
| --- | --- | --- | --- | --- |
| Neurofilament 200 (NF) | Mouse | Monoclonal IgG | 1:1000 | Sigma, St. Louis, USA (N0142) |
| Neurofilament 200 (NF) | Rabbit | Polyclonal IgG | 1:400 | Merck Millipore, Billerica, USA (N4142) |
| Synaptophysin (SYP) | Mouse | Monoclonal IgG | 1:200 | Sigma, St. Louis, USA (S5768) |
| glial fibrillary acidic protein (GFAP) | Mouse | Monoclonal IgG | 1:1000 | Abcam, London, UK (ab279289) |
| Postsynaptic density protein 95 (PSD95) | Rabbit | Polyclonal IgG | 1:800 | Abcam, London, UK (ab18258) |
| ionized Ca2+-binding adapter protein 1 (IBA-1) | Rabbit | Polyclonal IgG | 1:500 | Wako, Tokyo, Japan (SKN4887) |
| Microtubule-associated protein 2 (Map2) | Mouse | Monoclonal IgG | 1:1000 | Sigma, St. Louis, USA (M4403) |
| Choline acetyltransferase (ChAT) | Rabbit | Polyclonal IgG | 1:800 | Merck Millipore, Billerica, USA (AB2219) |
| c-Fos | Rabbit | Polyclonal IgG | 1:800 | Abcam, London, UK (ab209274) |
| Glutamic acid decarboxylase 1 (GAD67) | Rabbit | Monoclonal IgG | 1:500 | Abcam, London, UK (ab213508) |
| Laminin (LN) | Rabbit | Polyclonal IgG | 1:1000 | Sigma, St. Louis, USA (L9393) |
| paired box gene 7 protein (Pax7) | Mouse | Monoclonal IgG | 1:50 | DSHB, Douglas Houston, USA (Pax7) |
| Glial fibrillary acidic protein (GFAP) | Rabbit | Polyclonal IgG | 1:1000 | Boster, Wuhan, China (PB0046) |
| glyceraldehyde 3-phosphate dehydrogenase (GAPDH) | Mouse | Monoclonal IgG | 1:10000 | Abcam, London, UK (ab8245) |
| Vesicular glutamate transporters 1 (VGluT1) | Mouse | Monoclonal IgG | 1:2500 | Abcam, London, UK (ab242204) |
| Total OXPHOS cocktail | Mouse | Monoclonal IgG | 1:300 | Abcam, London, UK (ab110413) |
| Activity-regulated cytoskeleton-associated protein (Arc) | Mouse | Monoclonal IgG | 1:100 | Santa Cruz, Texas, USA (sc-166461) |
| Heat shock protein 90 (HSP90) | Rabbit | Monoclonal IgG | 1:5000 | Abcam, London, UK (ab203126) |
| ephrin A4 receptor (EphA4) | Mouse | Monoclonal IgG | 1:100 | Santa Cruz, Texas, USA (sc-135897) |
| Vesicular glutamate transporters 2 (VGluT2) | Rabbit | Monoclonal IgG | 1:5000 | Abcam, London, UK (ab216463) |
| microtubule-associated protein 2 (Map2) | Chicken | Polyclonal IgG | 1:1000 | Abcam, London, UK (ab5392) |
| Growth differentiation factor 8 (GDF8) | Mouse | Monoclonal IgG | 1:1000 | Abcam, London, UK (ab201954) |
| DyLigh 405 goat anti-rabbit secondary antibody | Goat | Polyclonal IgG | 1:500 | Jackson ImmunoResearch, West  Grove, USA (111-475-003) |
| Alexa 647 conjuncted goat anti-rabbit secondary antibody | Goat | Polyclonal IgG | 1:500 | Jackson ImmunoResearch, West Grove, USA (100699) |
| Cy3 conjuncted goat anti-rabbit secondary antibody | Goat | Polyclonal IgG | 1:300 | Jackson ImmunoResearch, West Grove, USA (711-165-162) |
| Cy3 conjuncted goat anti-mouse secondary antibody | Goat | Polyclonal IgG | 1:300 | Jackson ImmunoResearch, West Grove, USA (115-165-146) |
| [Goat anti-mouse HRP](http://www.abcam.com/goat-mouse-igg-hl-hrp-ab6789.html) | Goat | Polyclonal IgG | 1:5000 | Abcam, London, UK (ab6789) |
| Alexa fluor 647 goat anti-chicken secondary antibody | Goat | Polyclonal IgG | 1:5000 | Jackson ImmunoResearch, West Grove, USA (103-605-155) |
| [Goat anti-rabbit HRP](http://www.abcam.com/goat-mouse-igg-hl-hrp-ab6789.html) | Goat | Polyclonal IgG | 1:10000 | Abcam, London, UK (ab6721) |
| α-Bungarotoxin (BTX) conjugates alexa fluor 555 |  |  | 1:500 | Molecular Probe (B35451) |
