# Supplementary material for: Effects of tail nerve electrical stimulation on the activation and plasticity of the lumbar locomotor circuits and the prevention of skeletal muscle atrophy after spinal cord transection in rats
Source: CNS Neurosci Ther. 2023 Sep 26;30(3):e14445. doi: 10.1111/cns.14445 (PMC10916423; doi:10.1111/cns.14445)
Supplement: Supplementary file 18 — Data S1. [file CNS-30-e14445-s009.pdf]

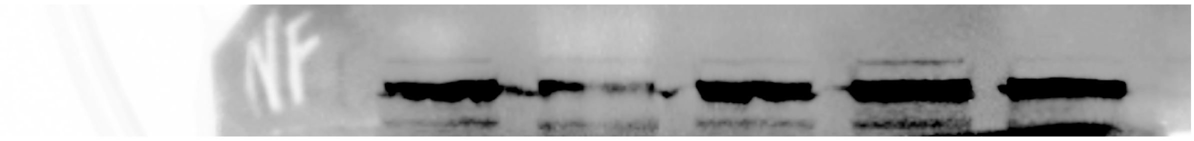

Full unedited blot for **Figure 3 J** (**NF:** 200 kDa)

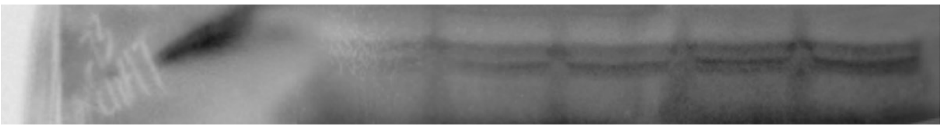

Full unedited blot for **Figure 3 J** (**ChAT:** 68 kDa)

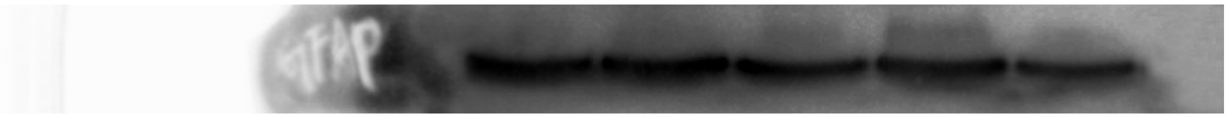

Full unedited blot for **Figure 3 J** (**GFAP:** 50 kDa)

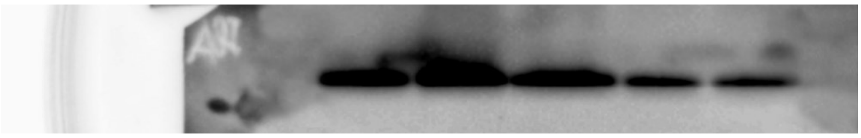

Full unedited blot for **Figure 3** (**IBA-1:** 17 kDa)

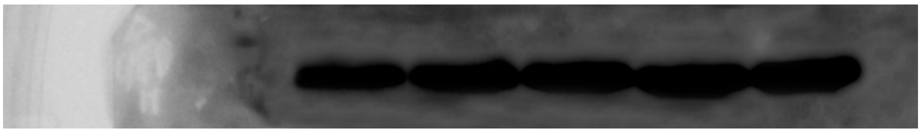

Full unedited blot for **Figure 3 J** (**GAPDH:** 37 kDa)

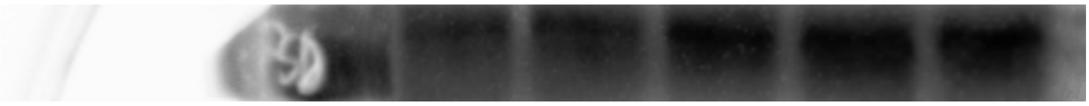

Full unedited blot for **Figure 5 G** (**PSD95**: 95 kDa)

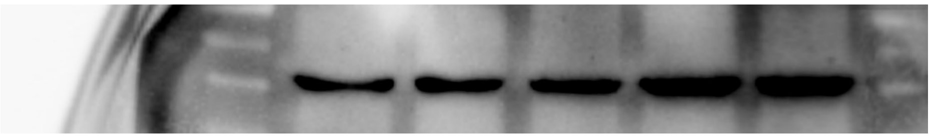

Full unedited blot for **Figure 5 G** (**GAD67**: 67 kDa)

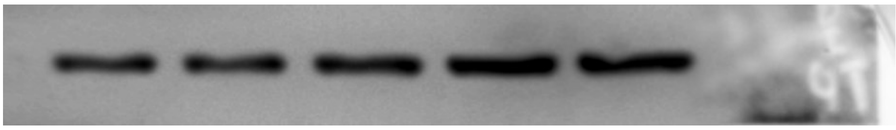

Full unedited blot for **Figure 5 G** (**VGluT1**: 62 kDa)

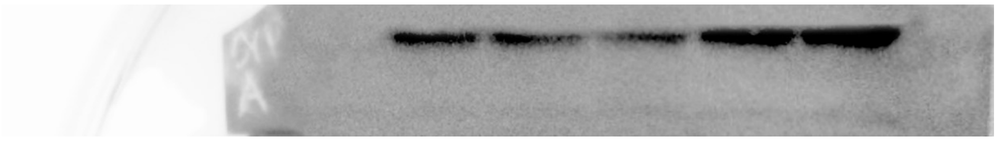

Full unedited blot for **Figure 5 G** (**SYP**: 38 kDa)

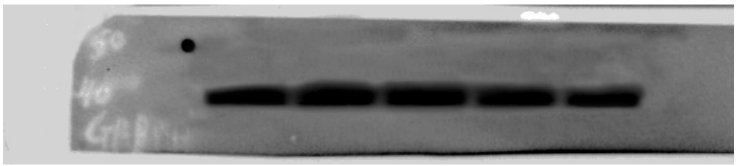

Full unedited blot for **Figure 5 G** (**GAPDH**: 37 kDa)

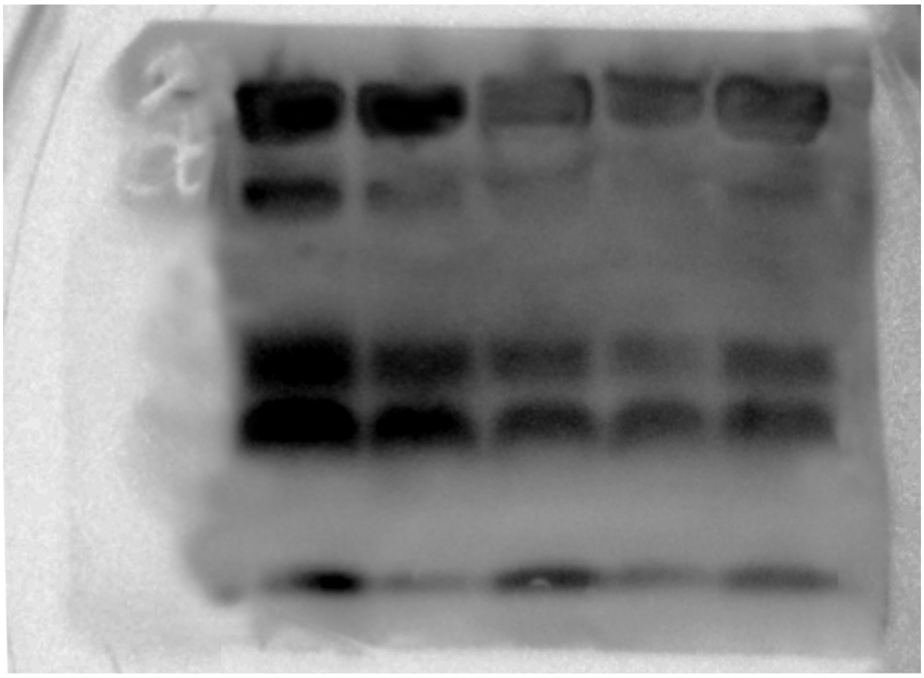

Full unedited blot for **Figure 6 E**, **Mitochondrial respiratory chain complex proteins** (**CV-APT5A**: 55 kDa; **CⅢ-UQCRC2**:48kDa; **CⅣ-MTCO1**: 40kDa; **CII-SDHB:** 30kDa; **CI-NDUFB8**: 20kDa)

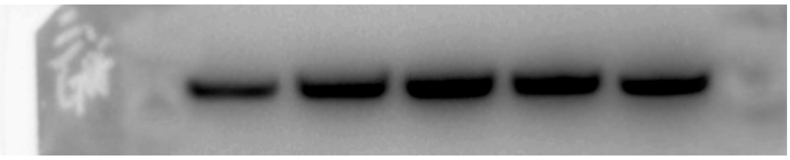

Full unedited blot for **Figure 6 E** (**GDF-8**: 43kDa)

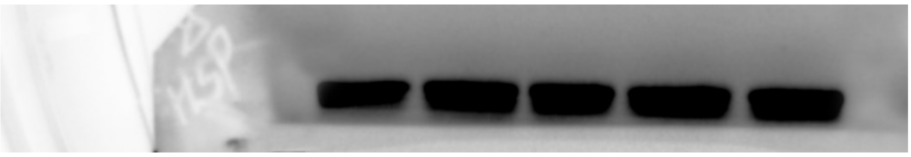

Full unedited blot for **Figure 6 E** (**HSP90**: 95kDa)
